# Mitochondrial dysfunction and lipid dysregulation in yeast lacking phosphatidylserine

**DOI:** 10.1091/mbc.E25-03-0128

**Published:** 2025-08-13

**Authors:** Alaumy Joshi, Zakery N. Baker, Rachel A. Stanfield, Dimitris T. Kalafatis, David J. Pagliarini, Vishal M. Gohil

**Affiliations:** ^a^Department of Biochemistry and Biophysics, Texas A&M University, College Station, TX 77843; ^b^Department of Cell Biology and Physiology, Washington University School of Medicine, St. Louis, MO 63110; ^c^Department of Biochemistry and Molecular Biophysics, Washington University School of Medicine, St. Louis, MO 63110; ^d^Department of Genetics, Washington University School of Medicine, St. Louis, MO 63110; ^e^Howard Hughes Medical Institute, Washington University School of Medicine, St. Louis, MO 63110; University of Gothenburg

## Abstract

Mitochondrial membrane phospholipids impact mitochondrial structure and function by influencing the assembly and activity of membrane proteins. Although the specific roles of the three most abundant mitochondrial phospholipids, phosphatidylcholine (PC), phosphatidylethanolamine (PE), and cardiolipin (CL), have been extensively studied, the precise function of less abundant phosphatidylserine (PS) is not yet determined. Here, we used genetic and nutritional manipulation to engineer a set of yeast mutants, including a mutant completely devoid of PS, to assess its role in mitochondrial bioenergetics and lipid homeostasis. To circumvent the confounding effect of downstream PS products, PE and PC, we exogenously supplied ethanolamine that allows their biosynthesis via an alternate pathway. Using this system, we demonstrate that PS does not impact the abundance or the assembly of mitochondrial respiratory chain complexes; however, mitochondrial respiration is impaired. PS-lacking mitochondria cannot maintain mitochondrial membrane potential and exhibit leaky membranes. A mass spectrometry-based analysis of the cellular and mitochondrial lipidomes revealed an unexpected increase in odd-chain fatty acid-containing lipids in PS-lacking cells that may impact mitochondrial bioenergetics. Our study uncovers novel roles of PS in mitochondrial membrane biogenesis and bioenergetics and provides a viable eukaryotic system to unravel the cellular functions of PS.

## Abstract (executive summary)

- Phospholipids determine the structure and function of mitochondria by influencing the biochemical and biophysical properties of their membranes.
- Previous studies have uncovered specific roles of abundant mitochondrial phospholipids, but the function of less abundant phospholipid - phosphatidylserine (PS) remains undetermined.
- We used yeast mutants in combination with nutritional supplementation to generate viable strains to study the role of PS.
- The biochemical, bioenergetic, and lipidomic profiling of these mutants revealed the essential requirement of PS for mitochondrial bioenergetics through its role in maintaining mitochondrial membrane potential and fatty acid composition.
- Our study provides a viable eukaryotic system to unravel the functions of PS.

## INTRODUCTION

Phospholipids are the major lipid constituents of protein-rich mitochondrial membranes (Zinser *et al.*, 1991; Horvath and Daum, 2013). Mitochondria contain all the major classes of phospholipids, with phosphatidylcholine (PC), phosphatidylethanolamine (PE), and cardiolipin (CL) being the most abundant, whereas phosphatidylserine (PS) and phosphatidic acid (PA) are present in very low amounts (Tuller *et al.*, 1999; Horvath and Daum, 2013). These phospholipids differ in their shape, size, and charge, imparting different biochemical and biophysical properties to the mitochondrial membranes (Joshi *et al.*, 2023). Over the last decade, studies with yeast phospholipid mutants have uncovered specific in vivo roles of each of the abundant mitochondrial phospholipids. For instance, PC has been shown to be required for the stability of the inner mitochondrial membrane translocase (TIM23) (Schuler *et al.*, 2016) and for the stability and function of the sorting and assembly machinery complex required for the insertion of β-barrel proteins in the outer mitochondrial membrane (Schuler *et al.*, 2016). PE has been shown to be required for the enzymatic activities of mitochondrial respiratory complexes (Bottinger *et al.*, 2012; Tasseva *et al.*, 2013; Baker *et al.*, 2016; Calzada *et al.*, 2019). CL, the signature phospholipid of mitochondria, has been implicated in mitochondrial protein import, mitochondrial dynamics, and bioenergetics (Basu Ball *et al.*, 2018; Falabella *et al.*, 2021; Ji and Greenberg, 2022). Specifically, CL has been shown to be essential for the assembly of mitochondrial respiratory chain (MRC) supercomplexes (Pfeiffer *et al.*, 2003; Zhang *et al.*, 2005) and ADP/ATP carrier (Claypool *et al.*, 2008; Senoo *et al.*, 2020) as well as for the stability of mitochondrial calcium uniporter (Ghosh *et al.*, 2020; Ghosh *et al.*, 2022) and mitochondrial magnesium channel (MRS2) (Joshi and Gohil, 2023).

The importance of the diversity of phospholipids and their nonoverlapping roles in mitochondrial physiology becomes apparent through rare human genetic disorders (Joshi *et al.*, 2023). For example, pathogenic mutations in the CL remodeling enzyme TAFAZZIN cause Barth syndrome, a debilitating disorder characterized by cardio-skeletal myopathy (Bione *et al.*, 1996). Mutations in the mitochondrial PE biosynthetic enzyme PISD cause Liberfarb syndrome, another devastating disorder characterized by developmental delay and neurological deficits (Girisha *et al.*, 2019; Peter *et al.*, 2019). A gain-of-function mutation in one of the PS biosynthesis enzymes, PSS1, causes a rare genetic disorder called Lenz–Majewski hyperostotic dwarfism (Sousa *et al.*, 2014).

**FIGURE 1:**
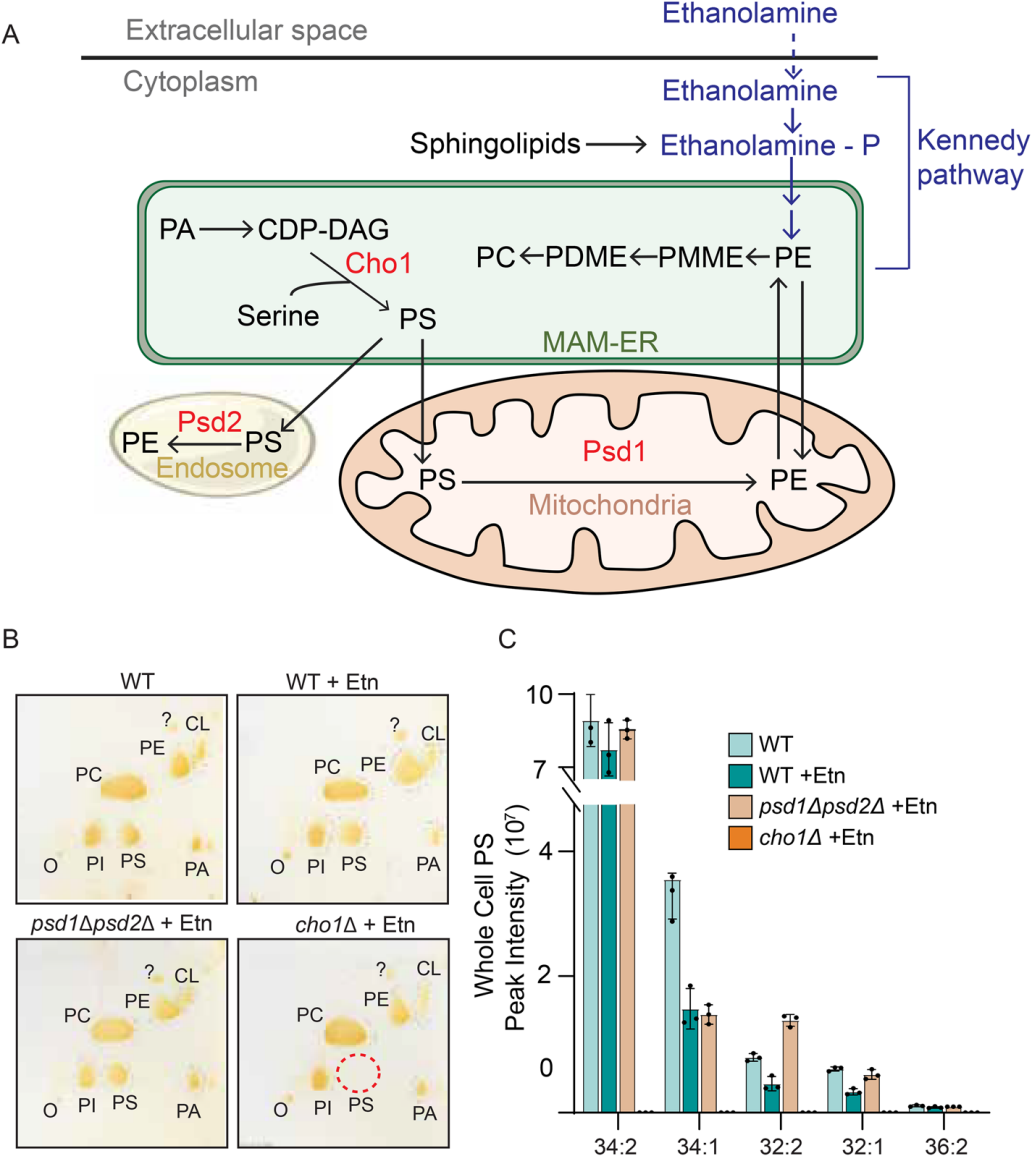
Yeast *S. cerevisiae* as a model system to study the in vivo role of PS. (A) Aminoglycerophospholipid biosynthetic pathways in yeast. PS is biosynthesized by condensation of CDP-DAG and serine in the ER via a reaction catalyzed by Cho1. PS is then transported to mitochondria or endosome, where it acts as a precursor for PE biosynthesis. *In situ* decarboxylation of PS by Psd1 and Psd2 generates PE in mitochondria and endosome, respectively. PE can also be biosynthesized in the ER via the Kennedy pathway from the endogenous pool of Etn phosphate generated from the breakdown of sphingolipids or the exogenously supplied Etn. PE undergoes sequential methylation reactions to form PC in the ER. Phospholipid exchange between the ER and mitochondria occurs at the mitochondria-associated ER membranes (MAM-ER). (B) An iodine-stained 2D TLC image of phospholipids extracted from the WT, and ethanolamine (Etn) supplemented WT, *psd1∆psd2∆*, *cho1∆* cells. The lack of a PS spot in *cho1∆* + Etn cells is outlined in a red circle. (C) Targeted lipidomic analysis of the PS in lipids extracted from the indicated yeast cells. PA, Phosphatidic acid; PI, Phosphatidylinositol; PS, Phosphatidylserine; PE, Phosphatidylethanolamine; CDP-DAG, Cytidine diphosphate diacylglycerol; PMME, Phosphatidylmonomethylethanolamine; PDME, Phosphatidyldimethylethanolamine; PC, phosphatidylcholine; CL, cardiolipin.

Unlike PE and CL, the mitochondrial and cellular functions of PS other than its well-described role in apoptosis have not been systematically determined (Vance and Steenbergen, 2005; Kay and Fairn, 2019). The major challenge in elucidating the in vivo functions of PS is the lack of a viable model system. Mice lacking both the PS biosynthetic enzymes PSS1 and PSS2 are not viable (Arikketh *et al.*, 2008). Moreover, PS is the biosynthetic precursor of PE, one of the most abundant phospholipids of the cell. Thus, deleting the PS biosynthetic enzyme is expected to disrupt PE biosynthesis as well, which in turn would affect PC biosynthesis because sequential methylation of PE generates PC in the endoplasmic reticulum (ER) (Figure 1A).

Previous studies have shown that yeast mutants lacking the PS biosynthetic enzyme, Cho1, can survive and grow when supplemented with ethanolamine (Etn), which allows PE biosynthesis via the Kennedy pathway (Atkinson *et al.*, 1980a; 1980b) (Figure 1A). Here, we took advantage of this redundancy in PE biosynthesis in yeast to construct a set of isogenic mutants to elucidate the specific role of PS in mitochondrial membrane biogenesis and bioenergetics.

## RESULTS

### A yeast system to study the in vivo role of mitochondrial PS

PS biosynthesis in yeast is carried out by a single enzyme, Cho1, which catalyzes the condensation of cytidine diphosphate diacylglycerol (CDP-DAG) with serine to form PS in the mitochondria-associated membranes of the ER (Figure 1A). Newly synthesized PS is then transported to mitochondria and endosomal compartments, where it serves as a substrate for PS decarboxylases Psd1 and Psd2, respectively, which convert it into PE (Figure 1A). Thus, deleting Cho1 not only eliminates PS biosynthesis but also de novo PE biosynthesis. It has been shown that the growth defect of *cho1∆* cells can be rescued with Etn supplementation, which allows PE biosynthesis via the Kennedy pathway (Atkinson *et al.*, 1980b; Birner *et al.*, 2001) (Figure 1A). However, *cho1∆* cells lack mitochondrial PE biosynthesis due to the absence of PS and Etn-derived PE cannot fully compensate for the loss of PE biosynthesized within mitochondria by Psd1 (Calzada *et al.*, 2019). Therefore, to specifically determine the role of PS in mitochondria it is necessary to compare Etn-supplemented *cho1∆* cells with a mutant that can biosynthesize PE only through the Etn-Kennedy pathway. To this end, we constructed the *psd1∆psd2∆* strain that like *cho1∆* is defective in mitochondrial PE biosynthesis, but when supplemented with Etn can biosynthesize PE by the Etn-Kennedy pathway (Baker *et al.*, 2016; Iadarola *et al.*, 2020; Iadarola *et al.*, 2021). Thus, a comparison of Etn supplemented *cho1∆* and *psd1∆psd2∆* would allow the determination of the specific role of PS in mitochondria. Toward this goal, we determined the phospholipid profile of wild-type (WT) and Etn-supplemented WT, *psd1∆psd2∆*, and *cho1∆* cells by two-dimensional (2D) thin layer chromatography (TLC) and found that *cho1∆* cells, as expected, do not contain any detectable PS, whereas all other phospholipids are present in all cell types (Figure 1B). We further confirmed the absence of various PS species in *cho1∆* cells by a targeted liquid chromatography-mass spectrometry (LC-MS)–based method (Figure 1C). In this manner, we generated four yeast strains that would allow us to determine the specific role of PS, as well as the contribution of mitochondrial and Kennedy pathway-generated PE to mitochondrial functions.

### Etn supplementation and loss of PS decarboxylation alter cellular and mitochondrial phospholipid profiles

Although TLC-based phospholipid analysis of yeast mutants provided a qualitative assessment of the major phospholipid classes, it is important to quantitatively determine various phospholipid species present in the mitochondria of our yeast mutants. This is critical because previous studies have shown that Etn supplementation cannot fully rescue MRC complex III activity (Calzada *et al.*, 2019) or the mitochondrial morphology defects (Chan and McQuibban, 2012) of *psd1∆psd2∆* mutants. This could be due to differences in PE species produced via the Etn–Kennedy pathway and the PS decarboxylation pathway or an inability for Etn–Kennedy pathway–derived PE species to efficiently transport to mitochondria. To explore these possibilities, we first performed untargeted LC-MS analysis of lipids extracted from the WT yeast cells cultured with and without Etn supplementation. We found that Etn supplementation increased the abundance of many PE species, as well as phosphatidylmonomethylethanolamine (PMME), and phosphatidyldimethylethanolamine (PDME) species that are intermediates of PC biosynthesis (Supplemental Figure S1A). This increase in cellular PE, PMME, and PDME is accompanied by a minor decrease in many PC species (Supplemental Figure S1A).

**FIGURE 2:**
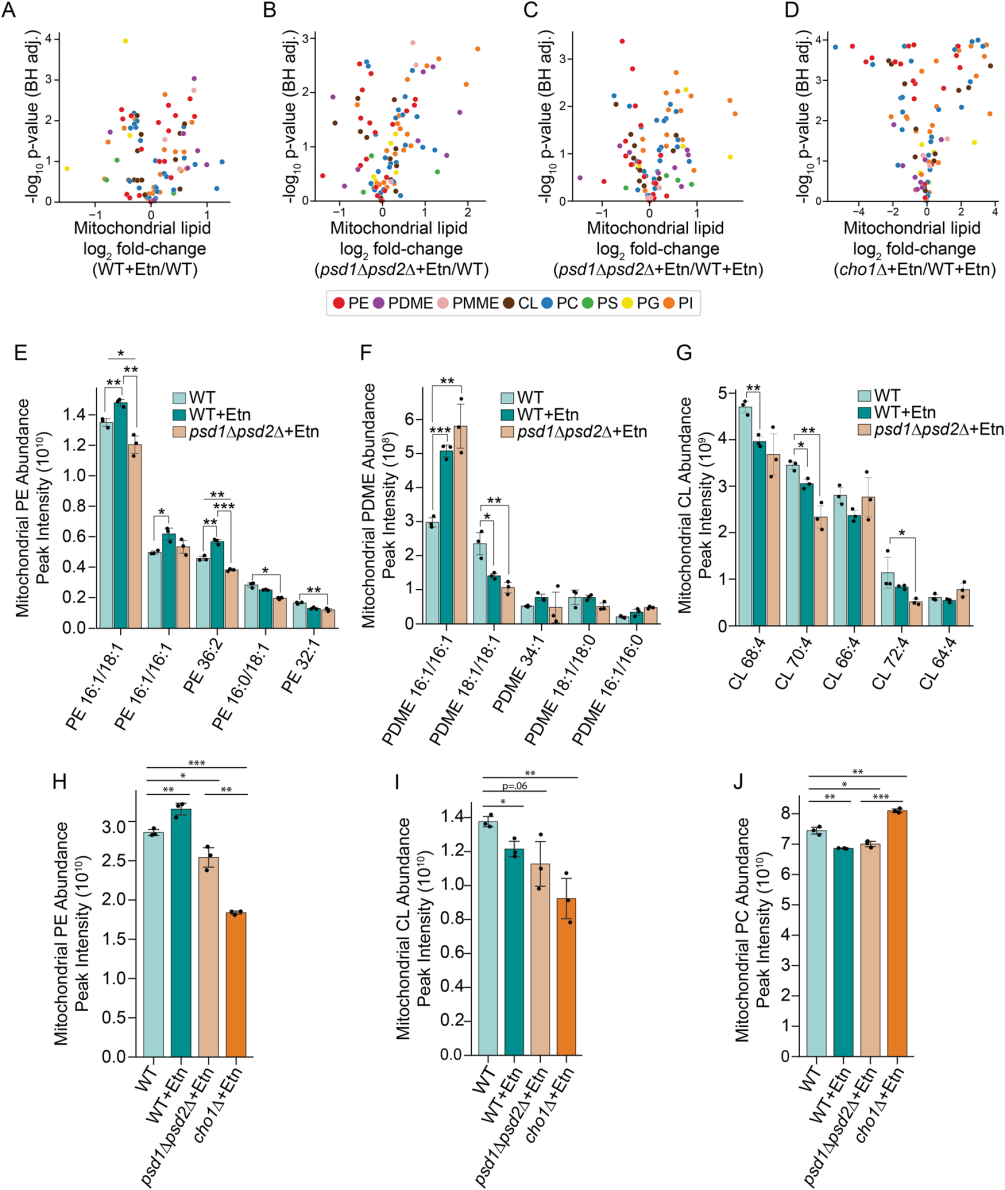
Ethanolamine supplementation alters the mitochondrial phospholipid profile. (A) Relative abundances (log_2_ fold-change) of select mitochondrial phospholipid species of WT yeast supplemented with Etn compared with WT versus statistical significance. (B) Relative abundances (log_2_ fold-change) of select mitochondrial lipid species of *psd1∆psd2∆* yeast supplemented with Etn compared with WT versus statistical significance. (C) Relative abundances (log_2_ fold-change) of select mitochondrial lipid species of *psd1∆psd2∆* yeast supplemented with Etn compared with WT supplemented with Etn versus statistical significance. (D) Relative abundances (log_2_ fold-change) of select mitochondrial lipid species of *cho1∆* yeast supplemented with Etn compared with WT supplemented with Etn versus statistical significance. (E–G) Normalized relative intensities for most abundant species of (E) PE, (F) PDME, and (G) CL in WT, WT supplemented with Etn and *psd1∆psd2∆* supplemented with Etn yeast. (H–J) Combined relative intensities of (H) PE, (I) CL, and (J) PC species in WT, WT supplemented with Etn, *psd1∆psd2∆* supplemented with Etn, and *cho1∆* supplemented with Etn yeast. For statistical significance, we have used Benjamini–Hochberg adjusted *P* value for panels A to D and two-sided *t* test for panels E to J. ***P < 0.001, **P < 0.01, *P < 0.05.

We then determined the phospholipid profiles of density gradient purified mitochondria from all four conditions and observed small but significant changes in the specific species of each of the major classes of phospholipids upon Etn supplementation in WT as well as different mutants (Figure 2, A–D). We first determined the impact of Kennedy pathway produced PE on the WT mitochondrial phospholipidome by comparing phospholipid profiles of WT mitochondria with mitochondria isolated from Etn-supplemented WT cells. We observed a small but significant increase in multiple highly abundant PE and PDME species along with reductions in the levels of the most abundant species of the mitochondria-specific phospholipid CL in mitochondria isolated from Etn-supplemented WT cells (Figure 2A, E–G). The presence of PDME in the mitochondrial extracts is likely due to residual ER contamination.

Next, we asked whether Etn supplementation restores the phospholipid profile of *psd1∆psd2∆* mitochondria to that of WT. To this end, we compared the phospholipid profiles of WT mitochondria with mitochondria isolated from *psd1∆psd2∆* cells cultured in the presence of Etn and found that many PE species were significantly lower in *psd1∆psd2∆* mitochondria (Figure 2E). We also observed an altered phospholipid profile in the whole-cell extract of WT and *psd1∆psd2∆* cells (Supplemental Figure S1B). Finally, we compared the phospholipid profiles of WT and *psd1∆psd2∆* cells when they both were cultured in the presence of Etn and found that the cellular and mitochondrial phospholipid profiles of these two cell types were different (Supplemental Figure S1C; Figure 2C, E–G). We see a similar perturbation in mitochondrial phospholipidome of PS-lacking *cho1∆* mitochondria (Figure 2D).

To compare overall change in the abundance of these phospholipid classes, we combined the intensities of individual PE, CL, and PC species for each condition and found a significant decrease in total PE levels in both Etn-supplemented *psd1∆psd2∆* and *cho1∆* mitochondria as compared with the WT mitochondria (Figure 2H). Notably, there was a more pronounced decrease in PE in *cho1∆* mitochondria as compared with *psd1∆psd2∆* (Figure 2H), suggesting that PS could be required for the mitochondrial import of Kennedy pathway PE. We also noticed a small but significant increase in total mitochondrial PE in Etn-supplemented WT cells, which can be due to increased PE biosynthesis via the Kennedy pathway (Figure 2H). Surprisingly, Etn supplementation reduced overall CL abundance in all strains (Figure 2I), which suggests a homeostatic regulation between PE and CL, the two nonbilayer forming phospholipids of mitochondrial membranes that have been shown to have overlapping functions (Gohil *et al.*, 2005; Joshi *et al.*, 2012). These perturbations in PE abundance triggered compensatory changes in PC levels as has been reported previously (Figure 2J) (Baker *et al.*, 2016; Basu Ball *et al.*, 2018).

Taken together, these results suggest that Etn supplementation alters cellular and mitochondrial phospholipidome and cannot fully restore the cellular or mitochondrial phospholipidome of *psd1∆psd2∆* cells. Thus, going forward it is important to compare *psd1∆psd2∆* with *cho1∆* to determine the specific role of PS in cellular and mitochondrial functions as both strains only contain PE derived from the Kennedy pathway.

### PS deficiency impairs respiratory growth without affecting the abundance and assembly of MRC complexes

**FIGURE 3:**
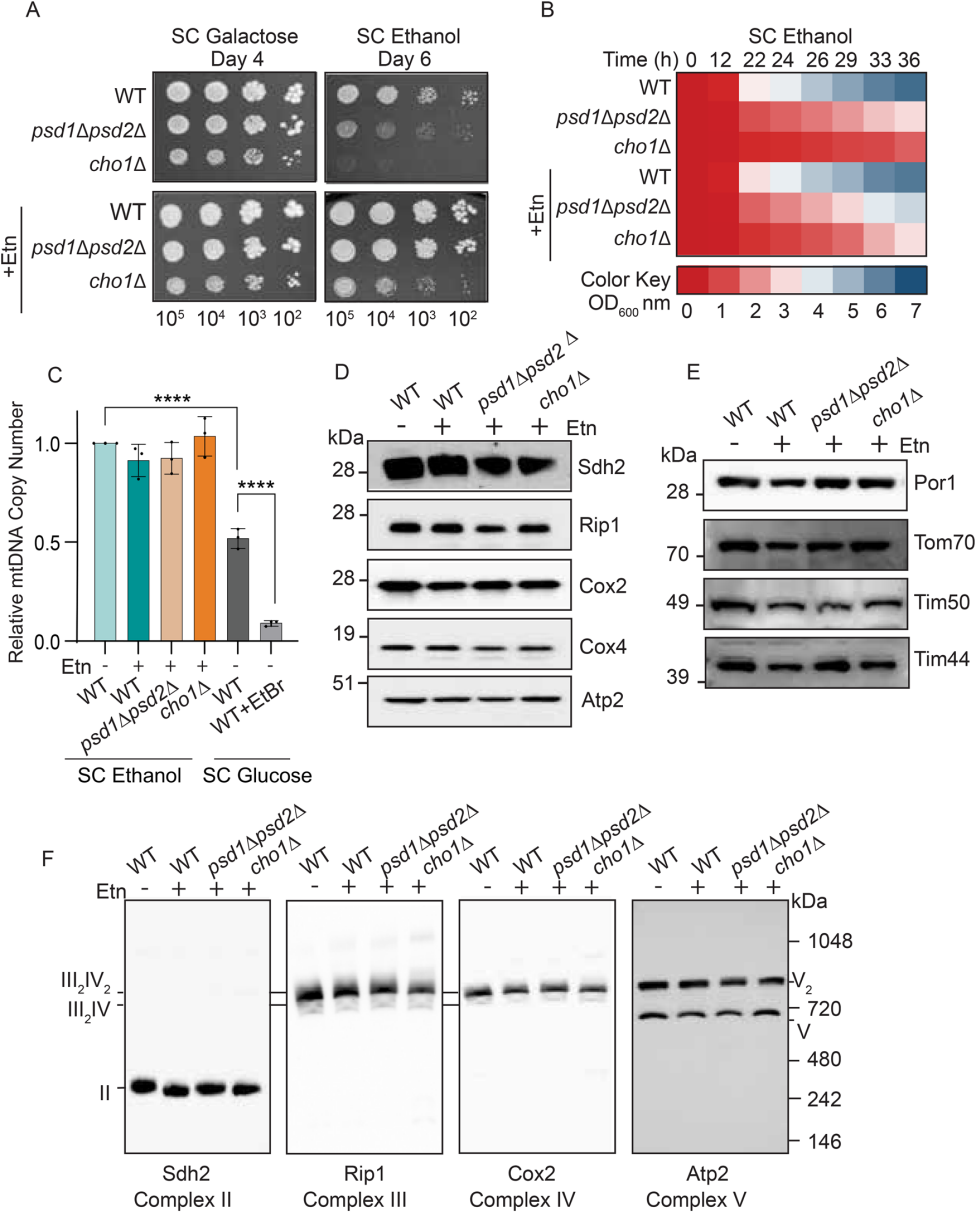
Respiratory growth is reduced in PS-lacking yeast cells. (A) Ten-fold serially diluted yeast cells were seeded onto SC media with the indicated carbon source with or without Etn supplementation and allowed to grow till the indicated time. Images were captured on day 4 for SC galactose and day 6 for SC ethanol grown cells. (B) Growth of WT, *psd1∆psd2∆,* and *cho1∆* cells at 30°C with or without Etn supplementation in SC ethanol liquid medium was monitored by measuring absorbance at 600 nm. (C) mtDNA copy number in the indicated yeast strains and growth conditions were measured by quantitative real-time PCR using *COX2 and POR1* TaqMan assays. Data are represented as mean ± SD (*n* = 3). ****P < 0.0001. (D) SDS–PAGE immunoblot-based detection of respiratory chain subunits of complex II (Sdh2), III (Rip1), IV (Cox2, Cox4), and V, (Atp2) in mitochondria isolated from the indicated yeast cells grown in SC ethanol medium. (E) SDS–PAGE immunoblot-based detection of outer and inner mitochondrial membrane proteins in mitochondria isolated from the indicated yeast cells grown in SC ethanol medium. (F) BN-PAGE immunoblot analysis of the digitonin-solubilized mitochondria isolated from the indicated yeast cells grown in SC ethanol medium. Complexes II–V were detected by Sdh2, Rip1, Cox2, and Atp2 antibodies, respectively. All panels in this figure are a representative of three biological replicates.

To determine the role of PS in mitochondrial respiratory functions, we first performed growth measurements of WT, *psd1∆psd2∆*, and *cho1∆* cells in the respirofermentative (SC Galactose) and respiratory (SC Ethanol) growth media with and without Etn supplementation. We found that *psd1∆psd2∆* cells grew like WT cells in respirofermentative medium, whereas *cho1∆* exhibited a mild growth defect in these conditions, suggesting a respiratory defect (Figure 3A). Growth in a strictly respiratory medium with only ethanol as a carbon source resulted in slow growth of *psd1∆psd2∆* and an almost complete lack of growth of *cho1∆* cells both of which were at least partially rescued with Etn supplementation (Figure 3A). We next performed a growth assay in liquid respiratory medium to get a more quantitative assessment of growth and found that Etn supplementation partially rescues reduced respiratory growth defects of *psd1∆psd2∆* and *cho1∆* cells (Figure 3B). Together, these results implied that in addition to PE, PS also plays a critical role in mitochondrial respiratory functions.

The reduced respiratory growth of *psd1∆psd2∆* and *cho1∆* cells could be due to a decrease in overall mitochondrial mass. Therefore, we measured mitochondrial DNA (mtDNA) copy number, a reliable indicator of mitochondrial mass and found no significant differences in any of the mutants (Figure 3C). The growth in glucose, which is known to suppress mitochondrial biogenesis in yeast, and treatment with ethidium bromide, which is known to inhibit mtDNA replication, resulted in an expected decrease in mtDNA copy number in the WT cells (Figure 3C). To ascertain the biochemical basis for reduced respiratory growth of PS-deficient cells, we analyzed the steady-state levels of denatured and native MRC complexes in the mitochondrial lysates from WT and Etn-supplemented WT, *psd1∆psd2∆*, and *cho1∆* cells grown in SC ethanol medium. We did not observe any marked changes in the protein abundance of individual subunits of the MRC complexes (Figure 3D) or the components of the mitochondrial protein import machinery (Figure 3E). The abundance and assembly of MRC complexes and supercomplexes were also not impaired in either of the mutants (Figure 3F). These results suggest that reduced respiratory growth of PE-depleted and PS-deficient cells is not due to the decreased abundance or assembly of the MRC complexes.

### PS is essential for maintaining mitochondrial membrane potential

**FIGURE 4:**
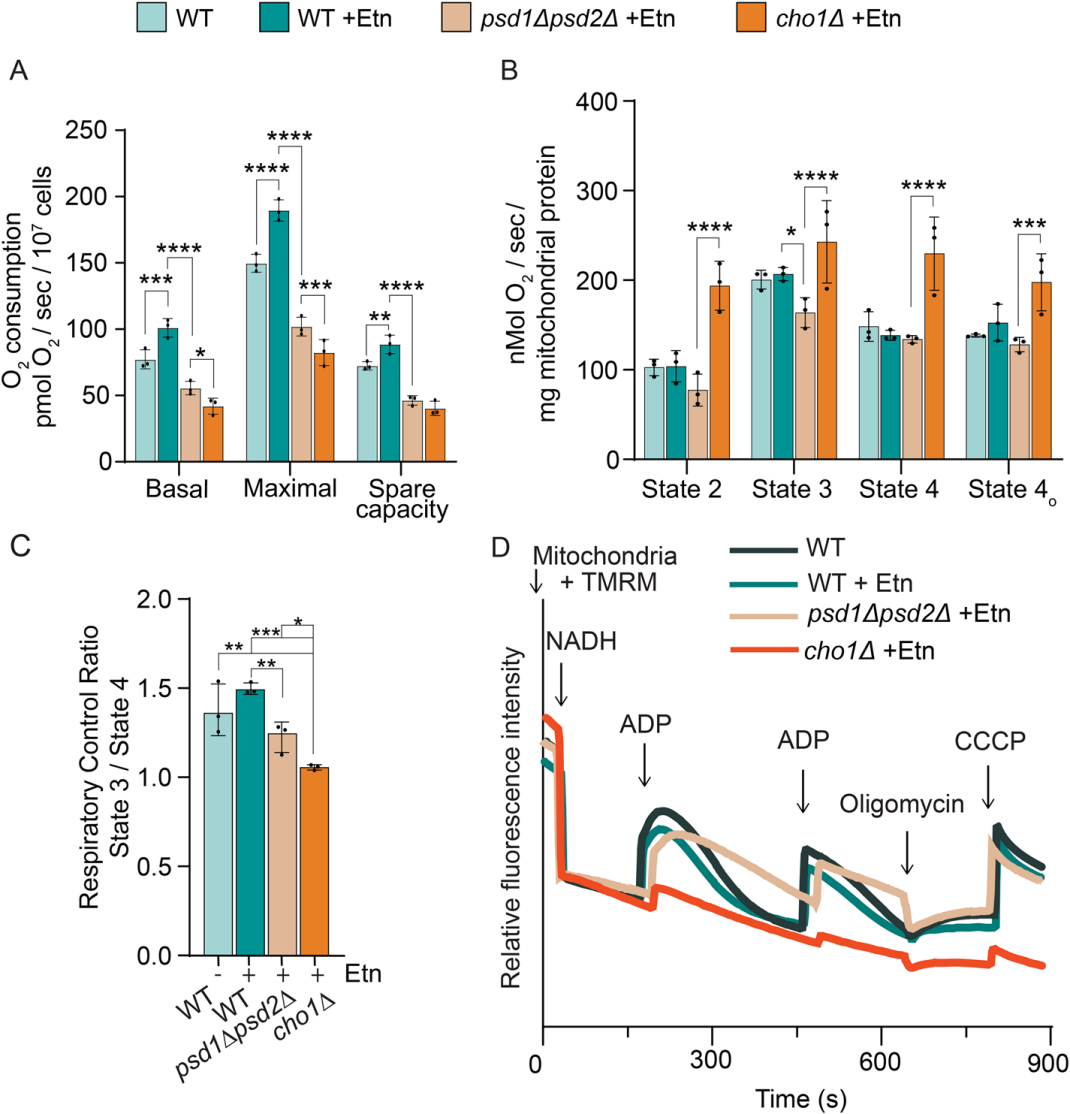
Mitochondrial bioenergetics in PS-lacking yeast. (A) Cellular OCR of indicated yeast cells measured at 30°C. Basal respiration refers to oxygen consumption before the addition of uncoupler, maximal respiration refers to oxygen consumption achieved after the addition of uncoupler CCCP, and spare respiratory capacity is calculated by subtracting basal respiration from maximal respiration rate. (B) States of mitochondrial respiration in the indicated yeast cells: NADH-driven oxygen consumption (state 2), ADP-stimulated oxygen consumption (state 3), resting respiration after ADP consumption (state 4), and oligomycin induced resting respiration (state 4_o_). Data are represented as mean ± SD (*n* = 3 biological replicates). (C) Respiratory control ratio (State 3/State 4) from samples analyzed in (B). Data are represented as mean ± SD (*n* = 3 biological replicates). (D) Representative TMRM traces from three independent experiments measuring the membrane potential (Δψ_m_) of mitochondria isolated from the indicated yeast strains. The Δψ_m_ was established by addition of 2 mM NADH followed by two sequential additions of 50 µm ADP to induce transient depolarizations. Oligomycin was used to hyperpolarize and CCCP used to depolarize mitochondria. ****P < 0.0001, ***P < 0.001, **P < 0.01, *P < 0.05.

Our previous study has shown that the reduced respiratory growth of *psd1∆* is due to reduced activity of MRC and not its decreased abundance (Baker *et al.*, 2016). Therefore, we measured the respiratory capacity of PE and PS mutants using a FluoRespirometer. Specifically, we measured basal oxygen consumption rate (OCR), uncoupler-stimulated maximal OCR, and spare respiratory capacity of WT and Etn-supplemented WT, *psd1∆psd2∆*, and *cho1∆* cells grown in SC ethanol medium. We found that the basal, maximal, and spare respiratory capacity of WT cells was slightly increased upon Etn supplementation (Figure 4A). Consistent with our respiratory growth assays (Figure 3, A and B), we found that basal and maximal OCR as well as the spare respiratory capacity of Etn-supplemented *psd1∆psd2∆* and *cho1∆* cells was significantly reduced when compared with the WT cells cultured in Etn (Figure 4A). When comparing respiration between *psd1∆psd2∆* and *cho1∆*, we observed a small but significant decrease in basal and maximal respiratory capacity of *cho1∆* cells (Figure 4A). To determine the extent of ADP-coupled respiration and proton leak in intact cells, we measured cellular respiration in the presence of oligomycin and found that unlike WT or the *psd1Δpsd2Δ* mutant, oligomycin treatment does not decrease respiration in *cho1Δ* cells (Supplemental Figure S2A), suggesting a high degree of proton leak in PS-lacking cells. These results imply that PE biosynthesized via Psd1 and Psd2 is essential for mitochondrial respiration and, independent of PE, PS is also critical for mitochondrial respiration.

To further dissect the role of PS in mitochondrial bioenergetics, we assessed the respiratory function of isolated mitochondria in all four conditions by measuring OCR during different states of respiration (Chance and Williams, 1955). We measured state 2 respiration by adding respiratory substrate (NADH) to isolated yeast mitochondria and surprisingly, found that PS-lacking mitochondria had 2-fold higher OCR (Figure 4B). We added ADP to isolated mitochondria to measure state 3 respiration, which measures the fraction of OCR that is used for ATP synthesis and is also called ADP-coupled respiration. The state 4 respiration was recorded when all the ADP was used up by mitochondria and it is attributed to the proton leak or the presence of any contaminating ATPases that recycle synthesized ATP to ADP. We found that PS-lacking *cho1∆* mitochondria exhibited higher state 4 respiration (Figure 4B), which could be attributed to a leaky IMM. To determine the contribution of proton leak to mitochondrial respiration, we measured state 4 respiration in the presence of oligomycin (state 4_o_), an inhibitor of ATP synthase, and found it to be also elevated in PS-lacking *cho1∆* mitochondria (Figure 4B). We observed similar trends with other respiratory substrates, including succinate and glutamate/malate, though we note that *cho1Δ* mitochondria exhibited more coupled respiration with these substrates as compared with NADH, with their respiration rates more comparable with WT conditions (Supplemental Figure S2, B and C). Consistent with these measurements, the respiratory control ratio (state 3/state 4) of *cho1∆* mitochondria was significantly reduced compared with mitochondria from all other strains (Figure 4C), which suggests that PS is required for efficient coupling of respiration with ATP synthesis. Notably, *psd1∆psd2∆* mitochondria exhibited only a small decrease in state 3 respiration and no significant change in state 2, state 4, or state 4_o_ respiration, implying that PE biosynthesized from PS decarboxylation is not essential for maintaining mitochondrial membrane permeability.

Reduced respiratory control ratio and increased state 4_o_ respiration of *cho1∆* mitochondria suggested increased proton leak through the IMM, which would result in reduced mitochondrial membrane potential (Δψ_m_). Therefore, we measured the Δψ_m_ of isolated mitochondria using the potentiometric fluorescent probe TMRM. We tracked the changes in Δψ_m_ following the addition of NADH, ADP, oligomycin, and an uncoupler (CCCP). NADH provides electrons for the MRC, increasing electron-coupled proton pumping across the IMM, hyperpolarizing the mitochondria, which was observed with mitochondria isolated from all four strains (Figure 4D). The addition of ADP to this system would cause a transient depolarization due to the utilization of the proton gradient to drive ATP production by ATP synthase (Complex V), resulting in an increased TMRM signal in the quench mode, as observed in the mitochondrial samples from all strains except *cho1∆,* which exhibited only a very small increase in TMRM signal (Figure 4D). The inner membrane is repolarized after all the ADP is consumed, which can be seen by a gradual decrease in TMRM fluorescence (Figure 4D). We found that the *psd1∆psd2∆* mitochondria repolarized with slower kinetics compared with WT mitochondria (Figure 4D), which could be attributed to reduced activity of complex V. The addition of complex V inhibitor oligomycin and uncoupler (CCCP) resulted in expected hyperpolarization and depolarization of IMM, respectively (Figure 4D). Treatment with CCCP and ADP showed that *cho1∆* mitochondria were unable to efficiently depolarize, which would indicate membrane leakiness and inability to use ADP. Taken together, these results suggest that a lack of mitochondrial PS makes mitochondrial membranes “leaky” resulting in the loss of membrane potential.

### PS-lacking yeast exhibit elevated levels of odd chain fatty acids

**FIGURE 5:**
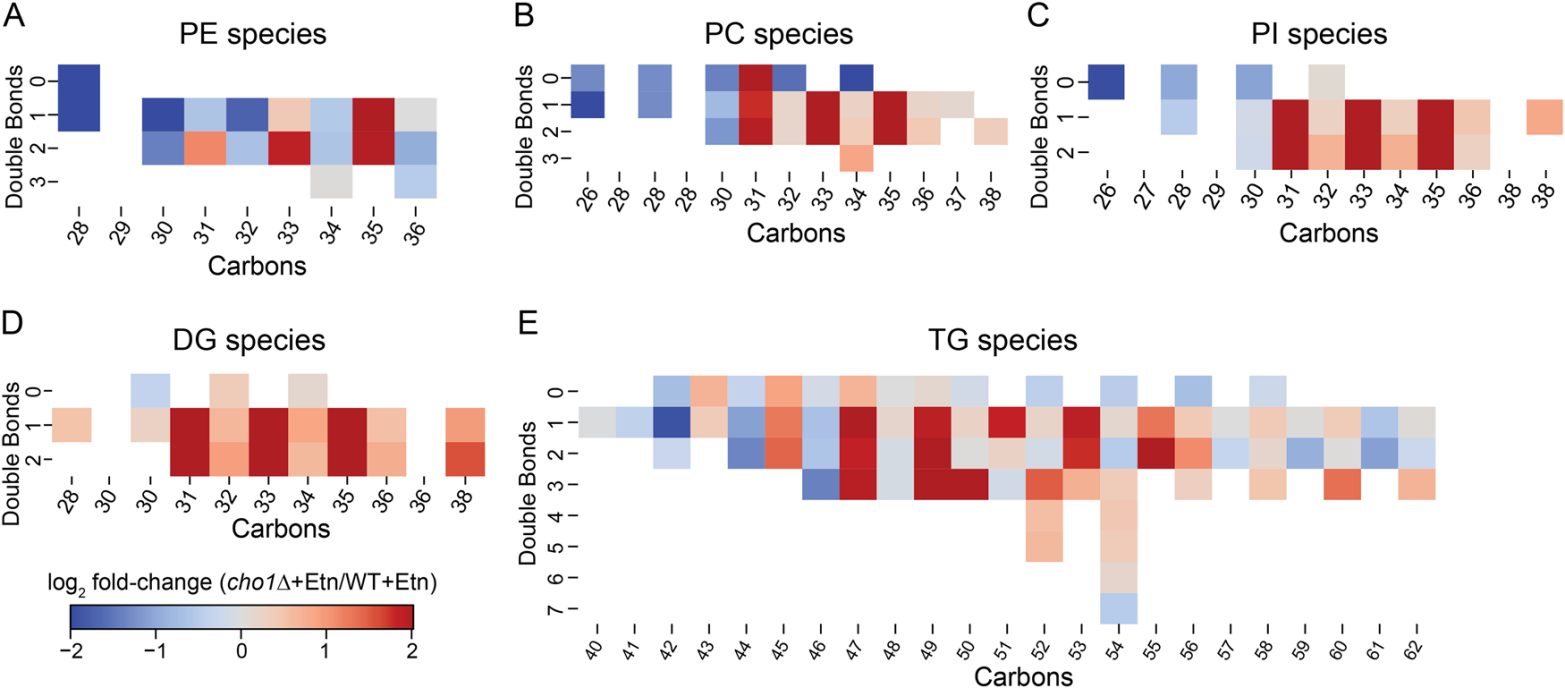
Loss of PS leads to increased odd chain fatty acids. (A–E) Heatmaps comparing carbon number and unsaturation in PE (A), PC (B), PI (C), diacylglycerol (DG) (D), and triacylglycerol (TG) (E) fatty acyl species between Etn-supplemented *cho1∆* and WT yeast mitochondria. The color bar represents the log_2_ fold change in lipid levels of *cho1∆* +Etn/WT +Etn.

Membrane biophysical properties such as fluidity and leakiness are influenced by changes in the saturation and the chain length of fatty acyl species (Budin *et al.*, 2018; Venkatraman *et al.*, 2024). Therefore, we analyzed the LC-MS lipidomics data of all four strains grown in the presence or absence of Etn. The comparison of the mitochondrial phospholipid profile showed a striking increase in the abundance of odd-chain fatty acid-containing phospholipids in *cho1∆* mitochondria (Figure 5, A–C). This change was not limited to phospholipids, as an increase in odd-chain fatty acyl species was also observed in diacylglycerols (DG) and triacylglycerols (TG) (Figure 5, D and E). Because the bulk properties of the membranes are determined by the overall abundance of individual lipids, we determined the relative fraction of the odd chain fatty acyl species to their even counterparts and found a small but significant increase in all lipid classes measured (Supplemental Figure S3). We also noticed minor changes in the degree of unsaturation of fatty acyl species in phospholipids and diacyglycerols in all Etn-supplemented conditions (Supplemental Figure S4). These results suggest that loss of PS triggers an increase in odd-chain fatty acid biosynthesis, which could influence biophysical properties of the mitochondrial membranes.

## DISCUSSION

Phospholipid environment is a strong determining factor in the activity, assembly, and stability of mitochondrial membrane proteins (Dowhan, 2017; Musatov and Sedlak, 2017). Previous studies have uncovered specific roles of CL, PE, and PC in mitochondrial structure and function but the role of PS has not been elucidated. This is mainly because PS is the precursor of more abundant mitochondrial phospholipids PE and PC, such that genetic ablation of its synthesis is also expected to disrupt the biosynthesis of PE and PC. Here, we overcome this limitation by stimulating a PS-independent pathway of PE biosynthesis with Etn supplementation in the yeast strains *cho1∆*, which lacks PS-biosynthetic enzyme, and *psd1∆psd2∆*, which lacks PS decarboxylases required for PE biosynthesis. Both these yeast strains utilize the same Etn–Kennedy pathway for PE biosynthesis from exogenously provided Etn, making the lack of PS biosynthesis in *cho1∆* cells the only difference. Using these strains in combination with WT cells allowed us to unravel PS-specific defects in mitochondrial bioenergetics and membrane biogenesis. Specifically, we found that PS is essential for maintaining Δψ_m_, a critical bioenergetic parameter that drives mitochondrial ATP synthesis (Figure 4D). Our work also uncovered an unexpected requirement of PS in fatty acid metabolism with the loss of PS leading to the increased levels of odd chain fatty acids in cellular and mitochondrial phospholipids and neutral lipids (Figure 5; Supplemental Figure S3).

PS is a negatively charged phospholipid that is more abundant in the plasma membrane, where it facilitates appropriate localization of proteins through charge-based interactions (Kay and Fairn, 2019). However, PS constitutes only ∼2% of the total phospholipids present in mitochondria (Horvath and Daum, 2013). The bulk of PS imported from the mitochondria-associated membranes of the ER is converted to PE by PS decarboxylase within mitochondria (Figure 1A). Thus, PS is not expected to significantly contribute to the biogenesis of mitochondrial membrane proteins or the bulk physical properties of the membrane. Consistent with this idea, we found that loss of PS did not impact the abundance or the assembly of mitochondrial membrane proteins and complexes (Figure 3, D–F). However, high uncoupled respiration (Figure 4, B–C; Supplemental Figure S2A) in PS-deficient cells was surprising and is indicative of functional MRC but a leaky IMM. This was confirmed by Δψ_m_ measurements, where PS-deficient mitochondria were unresponsive to agents that would depolarize or hyperpolarize mitochondria (Figure 4D). These findings are consistent with recent studies in a *Drosophila* model of PS deficiency, where the knockdown of PS biosynthetic enzyme in the salivary gland cells of larvae resulted in loss of mitochondrial membrane integrity (Yang *et al.*, 2019). The molecular basis for this aspect of PS function remains to be elucidated.

In addition to elucidating the role of PS in mitochondrial biology, our study also allowed us to determine the roles of different PE species produced via the Etn–Kennedy pathway and the PS decarboxylation pathway. First, we found that stimulating the Etn–Kennedy pathway by exogenous Etn supplementation results in a distinct cellular and mitochondrial phospholipid profile with an increased abundance of multiple PE and PDME species (Figure 2, A, E, and F). Second, PE species produced by the Etn–Kennedy pathway are different than the PE species produced by the PS decarboxylation pathway (Figure 2B; Supplemental Figure S1B), a finding consistent with what has been observed in mammalian cells (Bleijerveld *et al.*, 2007). Third, the incorporation of Etn–Kennedy pathway PE species into mitochondria alters the abundance of other mitochondrial phospholipid species, including CL, PI, and PC (Figure 2A). This difference in PE and other phospholipid species could explain the incomplete rescue of cellular respiration in *psd1∆psd2∆* by Etn supplementation (Figure 4A). Additionally, in Etn-supplemented *psd1∆psd2∆* and *cho1∆* mitochondria, the overall abundance of PE and CL, phospholipids critical for MRC activity and assembly, were reduced (Figure 2, H and I). Interestingly, PE levels in *cho1∆* mitochondria were further reduced when compared with *psd1∆psd2∆*, which suggests that PS could play a role in the import of PE from the ER (Figure 2H). This reduction in PE could be a critical contributor of mitochondrial bioenergetic defects observed in PS-lacking *cho1∆* cells (Figure 2H). Notably, we had previously reported that PE synthesized via the Etn–Kennedy pathway is able to fully rescue respiration of *psd1∆* cells even though it is not efficiently incorporated into mitochondria (Baker *et al.*, 2016). Our studies with *psd1∆psd2∆* cells imply that endosomal PE biosynthesized by Psd2 is also transported into *psd1∆* mitochondria where it contributes to mitochondrial respiration.

Finally, untargeted lipidomic analysis of these yeast mutants led to a completely unexpected discovery that PS deficiency triggers the accumulation of odd-chain fatty acids in phospholipids and neutral lipids (Figure 5; Supplemental Figure S3). Typically, odd-chain fatty acids are produced from propionyl-CoA, but due to the low production of intracellular propionyl-CoA, these are not commonly found in cells and tissues. Propionyl-CoA can be synthesized from branched-chain amino acids like valine, isoleucine, threonine, and methionine (Park *et al.*, 2019; Ma *et al.*, 2023) or by α-oxidation of even chain fatty acids (Fulco, 1967; Foulon *et al.*, 2005). Currently, we do not know which of these pathways contribute to elevated odd-chain fatty acids in PS-deficient cells. Future studies are needed to delineate these pathways and underlying mechanisms.

In summary, our studies have established a facile genetic model system to interrogate cellular functions of PS, a ubiquitous phospholipid found in eukaryotic cellular membranes. Recently, we have used our isogenic yeast phospholipid mutants of PE, PC, and CL biosynthetic pathways as surrogate models to determine the specific phospholipid requirements of human proteins (Ghosh *et al.*, 2020; Ghosh *et al.*, 2022; Joshi and Gohil, 2023). We envision that mutants presented in this study will be similarly useful in identifying PS-requirements for human proteins.

## MATERIALS AND METHODS

Request a protocol through *Bio-protocol*

The yeast strains used in this study are derived from *Saccharomyces cerevisiae* WT strain BY4741 (*MATa, his3Δ1, leu2Δ0, met15Δ0, ura3Δ0*), which is a S288C-derivative laboratory strain. They are listed in Supplemental Table S1.

### Yeast growth conditions

Yeast cells were maintained in complex media [1% (wt/vol) yeast extract, 2% (wt/vol) peptone, with 3% (vol/vol) glycerol and 1% (vol/vol) ethanol as the carbon sources. For growth measurements, yeast cells were grown in synthetic complete (SC) media [0.17% (wt/vol) yeast nitrogen base, 0.5% (wt/vol) ammonium sulfate, 0.2% (wt/vol) amino acid mix], with either 2% (vol/vol) ethanol, 2% (wt/vol) galactose, or 2% (wt/vol) glucose as a source of carbon. Solid agar media were prepared by further adding 2% (wt/vol) agar. Etn (2 mM) was added into the growth media wherever indicated. All strains were grown at 30°C. Liquid cultures were grown in an incubator-shaker at 250 rpm. Yeast growth measurement on agar plate was performed by seeding 3 µl of 10-fold serial dilutions of preculture onto the indicated plates. The images for the plates were taken after 4 d of growth in SC galactose- and 6 d of growth on SC ethanol-medium. For growth measurements in the liquid media, the cultures were started at an optical density of 0.1 at 600 nm and the optical density was measured at the indicated timepoints to observe growth at 30°C.

### Quantitative real-time PCR analysis

Yeast cells were grown to mid-log phase in either SC ethanol or SC glucose media. To induce mtDNA depletion, 25 µg/ml ethidium bromide (EtBr) was added in SC glucose medium. DNA was isolated with glass bead beating using the QIAGEN Plasmid Plus Midi Kit. The *C*_t_ values for a nuclear gene (*POR1*) and a mitochondrial-DNA–encoded gene (*COX2*) were measured by using quantitative real-time PCR performed in a 96-well plate. A total of 20 µL of PCR samples were prepared using 9 µL of DNA, 10 µL of mastermix, and 1 µL of TaqMan assay (*COX2: Sc04164581_s1, POR1: Sc04157843_s1*, Thermo Fisher Scientific). Mitochondrial copy number was quantified using the ∆∆*C*_t_ method.

### Mitochondria isolation

Crude mitochondria were isolated by subcellular fractionation as described previously (Meisinger *et al.*, 2006). The yeast cells were spheroplasted using zymolyase followed by lysis using a glass homogenizer. The crude mitochondrial preparation was obtained after sequential differential centrifugation at 1500, 4000, and 12,000 × *g*. The crude mitochondrial fraction was either used fresh for mitochondrial respiration measurements or further processed to obtain highly pure mitochondrial preparation. Crude mitochondria were loaded on 60, 32, 23 and 15% sucrose gradient, followed by density gradient centrifugation at 100,000 × g for 1 h at 4°C. After centrifugation, the highly pure mitochondrial fraction was carefully extracted from the interface of 60 and 32% sucrose and washed with buffer containing 250 mM sucrose, 1 mM EDTA and 10 mM MOPS and pelleted at 12,000 × g. The pure mitochondria were flash frozen at –80°C till further use.

### SDS–PAGE, BN-PAGE, and immunoblotting

Denatured and native yeast mitochondrial protein lysates were separated using SDS–PAGE and Blue Native PAGE (BN-PAGE), respectively. For SDS–PAGE, mitochondrial lysates were prepared using NuPAGE LDS sample buffer (4X) and NuPAGE sample reducing agent (10X) (Thermo Fisher Scientific, Carlsbad, CA) and 20 µg of mitochondrial protein were resolved on 10 or 12% NuPAGE Bis-Tris gels (Thermo Fisher Scientific, Carlsbad, CA). Separated proteins were subsequently transferred onto polyvinylidene fluoride (PVDF) membranes using a Trans-Blot SD semidry transfer cell (Bio-Rad, Hercules, CA). In the case of BN-PAGE, mitochondria were solubilized in 4X Native PAGE sample buffer containing 1% digitonin at a concentration of 6 g/g of mitochondrial protein, followed by incubation on ice for 15 min. The resulting lysate was centrifuged at 20,000 × g for 30 min, and the clear supernatant containing the native complexes was collected. To this, 50x G-250 sample additive was added. Twenty micrograms of the native protein lysate were subsequently resolved on a 4 to 16% Native PAGE Bis-Tris gel and transferred onto a PVDF membrane using a wet transfer method. Following transfer, the PVDF membranes were blocked for 1 h in TBS with 0.1% Tween 20 (TBST) containing 5% nonfat dry milk powder. The blots were then probed with primary antibodies at 4°C overnight, followed by incubation with secondary antibodies for 1 h at room temperature. All the antibodies used in this article are listed in Supplemental Table S2. Finally, the membrane was developed using Clarity or Clarity Max ECL Western blotting substrates (Bio-Rad Laboratories) on a Bio-Rad ChemiDoc MP imaging system.

### Cellular respiration measurements

Total cellular OCR was measured using high-resolution O2K FluoRespirometer (Oroboros) as described previously (Iadarola *et al.*, 2021). O2K chamber was set to 30°C with stirring at 250 rpm. OCR measurements were performed in 2 × 10^7^ cells in 2 ml of SC-ethanol in the O2K chamber. After measuring the basal respiration, 5 µM carbonyl cyanide m-chlorophenyl hydrazone (CCCP) was added into the chamber to determine maximal respiration. Nonmitochondrial respiration was measured after injecting 1 µM antimycin A. Spare respiratory capacity was determined by calculating the difference between basal and maximal respiration. Non-ATP–dependent respiration was measured by treating cells with oligomycin (3 µg/ml) for 3 h.

### Mitochondrial bioenergetic assays

Measurements of states of respiration and assessment of mitochondrial membrane potential (Δψ_m_) were performed from freshly isolated mitochondria using the high-resolution O2K FluoRespirometer (Oroboros) and following the protocol adapted from (Baile *et al.*, 2014; Kohler *et al.*, 2023). For these measurements, the O2k–FluoRespiratory module is operated through the Amperometric (Amp) channel. Freshly isolated crude mitochondrial samples were resuspended in measurement buffer (MB) containing 20 mM Tris-HCl, pH 7.2, 20 mM KCl, 3 mM MgCl_2_, 4 mM KH_2_PO_4_, and 250 mM sucrose for measuring oxygen consumption and Δψ_m_. The O2K chamber was prepared by adding 2 ml of MB and chamber temperature was set to 30°C with stirring at 250 rpm, in Amp channel: “Gain for Fluo sensor” and “Fluo intensity” were both set to 1000, Fluorescence-Sensor Green Filter Set AmR was used for fluorescence measurements. During these measurements the chamber illumination was turned off. For Δψ_m_ measurements, 0.5 µM of lipophilic cationic dye tetramethylrhodamine methyl ester (TMRM) was added to the chamber and was used in the quenched mode (i.e., its fluorescence signal is quenched when large amount of the dye is accumulated in the mitochondrial matrix). TMRM accumulate into the mitochondrial matrix in a manner proportional to Δψ_m_, according to the Nernst equation, and its redistribution is consistent with the changes in Δψ_m_. The measurements were recorded from 100 µg of crude mitochondria after the fluorescence and respiration signal is stabilized in the instrument. Following respiratory substrates and inhibitors/uncouplers were used to assess various states of mitochondrial respiration and changes in Δψ_m_. To the 100 µg of crude mitochondria, 40 µl of 0.1 M NADH (final concentration of 2 mM), 10 µl of 2 M glutamate (final concentration 10 mM), and 10 µl of 400 mM malate (final concentration 2 mM), or 20 µl of 1M succinate (final concentration of 10 mM), was added to record the state 2 respiration. Next, 1 µl of 0.1 M ADP (final concentration of 50 µM) was added to the chamber to measure ADP-coupled respiration (state 3). The second pulse of ADP was added 5 min after the first pulse. Upon consumption of ADP by respiring mitochondria, respiratory rate drops which is recorded as state 4 respiration. To measure the state 4_o_ respiration, 0.5 µl of 10 mM oligomycin (final concentration of 2.5 µM) was added. Subsequently, 0.5 µl of 20 mM CCCP (final concentration of 5 µM) was added to determine the maximal respiratory capacity. Respiratory control ratio was determined by calculating state 3/state 4 respiratory rates. Traces of changes in membrane potential were recorded and plotted using GraphPad Prism. The source and catalogue number of all chemicals used are listed in Supplemental Table S2.

### Lipid extraction and TLC

Whole-cell phospholipids were extracted using Folch extraction method as described previously (Iadarola *et al.*, 2021). Briefly, lipids were extracted from spheroplasts with Folch solution (2:1 chloroform:methanol). The extracted lipids were first washed with water followed by another wash with 1:1 water:methanol and were then dried under nitrogen gas. Dried phospholipids were resuspended in 100% chloroform and were separated by 2D-TLC on TLC Silica gel 60 aluminum plates (Millipore Sigma) using the following solvent system: chloroform/methanol/ammonium hydroxide (32.5/17.5/2.5) in the first dimension, followed by chloroform/acetic acid/methanol/water (37.5/12.5/2.5/0.7) in the second dimension. Phospholipids were visualized with iodine vapor.

### Lipid extraction for mass spectrometry-based lipidomics

Frozen cell pellets were first resuspended in 225 µl of 100% LC-grade methanol (Thermo Fisher Scientific) containing 1 µM CoQ_8_ as an internal standard. Glass beads (100 µl; 0.5 mm; BioSpec) were then added and the samples were vortexed for 10 min (3000 rpm, 4°C) to lyse the cells. A total of 187.5 µl of LC-MS grade water (Sigma-Aldrich) and 750 µl of methyl tert-butyl ether (MTBE) (Sigma-Aldrich) were added to each sample and tubes were vortexed again for 3 min (3000 rpm, 4°C). To separate layers, samples were centrifuged for 3 min (1000 × *g*, 4°C). Organic (top) layer was removed into a separate microcentrifuge tube and a new 750 µl of MTBE was added. Organic extraction was repeated a second time with the second MTBE layer added to the first. Samples were dried by vacuum centrifugation and resuspended in 50 µl of 20 mM ammonium acetate in 78% (vol/vol) methanol, 20% isopropyl alcohol (Sigma-Aldrich), 2% water.

### LC-MS–based untargeted lipidomics

LC-MS was performed using a Vanquish Binary Pump (Thermo Fisher Scientific) coupled to a Thermo Exploris 240 Orbitrap mass spectrometer. A total of 2 µl of extracted lipids were injected by the autosampler and separated using an Acquity CSH C18 column (100 mm × 2.1 mm × 1.7 µm particle size; Waters) kept at 50°C. For separation mobile phase A consisted of 10 mM ammonium acetate (Sigma-Aldrich) in 70% (vol/vol) acetonitrile, 30% (vol/vol) water with 250 µl/L acetic acid (Sigma-Aldrich). Mobile phase B consisted of 10 mM ammonium acetate in 90% (vol/vol) isopropyl alcohol 10% (vol/vol) with 250 µl/L acetic acid. The following gradient was used: 2% mobile phase B from 0 to 2 min, increased to 30% B over next 1 min, increased to 50% B over next 1 min, increased to 85% over next 14 min, increased to 99% B over next 1 min, then held at 99% B for next 7 min (400 µl/min flow rate). Column reequilibration of 2% B for 1.75 min occurred between samples.

Samples were ionized by a HESI II source (Thermo Fisher Scientific) kept at the following parameters: vaporizer temperature 350°C, sheath gas 50 U, auxiliary gas 8 U, and sweep gas 1 U. The MS was operated in polarity switching mode with the spray voltage set to 3500 V for positive mode and 2500 V for negative mode. The inlet ion transfer tube temperature was kept at 325°C with 70% RF lens. For discovery based untargeted analyses, full MS1 scans were acquired at 22,500 resolution (at 200 m/z), max ion accumulation time of 100 ms, with a scan range of m/z 200 to 1600. MS2 scans (Top 3) were acquired at 30,000 resolution (at 200 m/z), max ion accumulation time of 50 ms, 1.0 m/z isolation window, stepped normalized collision energy at 20, 30, 40, and a 10.0 s dynamic exclusion. Automatic gain control targets were set to standard mode for both MS1 and MS2 acquisitions.

LC-MS files for lipidomics were processed using Compound Discoverer 3.1 (Thermo Fisher Scientific). Peaks with retention times between 1.4 and 23 min and having 100 Da to 5000 Da were retained. Ten ppm mass tolerance and 0.4 min retention time tolerance filters were used. To be included, peaks had to have an intensity greater than 2 × 10^6^, width less than 0.75 min, signal-to-noise ratio less than 1.5, and intensity 5-fold greater than blank. MS2 spectra were searched using LipiDex 1.0 (Hutchins *et al.*, 2018) against an in-silico generated spectral library (Hutchins *et al.*, 2019) with the following parameters: dot product score > 500, and reverse dot product score > 700. Lipid MS/MS spectra that contained <75% interference from coeluting isobaric lipids and eluted within a 3.5 median absolute retention time deviation of each other were used for identification. Lipid identifications were filtered by retention time modeling using LipidDex2 Degreaser module (v0.1.0) (Anderson *et al.*, 2024). The retention time tolerance used was 0.5 min.

### LC-MS–targeted lipidomics

For targeted PS analyses, chromatography conditions were the same as untargeted analysis. The mass spectrometer was operated in parallel reaction monitoring mode with high-energy collisional dissociation at 30%, targeting selected ion fragments generated from the fragmentation of the loss of hydrogen (H^−^) ion. List of targeted compounds, precursor m/z, targeted fragment m/z and retention times are given in Supplemental Table S3. Select fragments were chosen for quantification based on previously published fragmentation patterns for PS (Pi *et al.*, 2016). Peak integration was performed using Tracefinder 5.1 (Thermo Fisher Scientific).

### Data quantification and statistical analysis

Statistical analysis of lipidomics data was performed by Student's *t test* or one way ANOVA using MetaboAnalyst 6.0. (https://www.metaboanalyst.ca/MetaboAnalyst/). Data are shown as mean ± SD (SD), and the number of replicates is indicated in the figure legends. A *P* value of <0.05 was considered statistically significant. Statistical analysis for all other experiments were performed using GraphPad Prism software version 10.1.2 (GraphPad Software, La Jolla, CA, https://www.graphpad.com).

## Supporting information





## Abbreviations used:

CDP-DAG: cytidine diphosphate diacylglycerol
CL: cardiolipin
DG: diacylglycerol
Etn: ethanolamine
PA: phosphatidic acid
PC: phosphatidylcholine
PDME: phosphatidyldimethylethanolamine
PE: phosphatidylethanolamine
PI: phosphatidylinositol
PMME: phosphatidylmonomethylethanolamine
PS: phosphatidylserine
TG: triacylglycerol.
