# Supplementary material for: Mitochondrial dysfunction and lipid dysregulation in yeast lacking phosphatidylserine
Source: Mol Biol Cell. 2025 Aug 13;36(10):ar121. doi: 10.1091/mbc.E25-03-0128 (PMC12444905; doi:10.1091/mbc.E25-03-0128)
Supplement: Supplementary file 1 [file mbc-36-ar121-s001.pdf]

Supplemental Materials

Molecular Biology of the Cell

Joshi *et al*.

Supplementary Figure 1

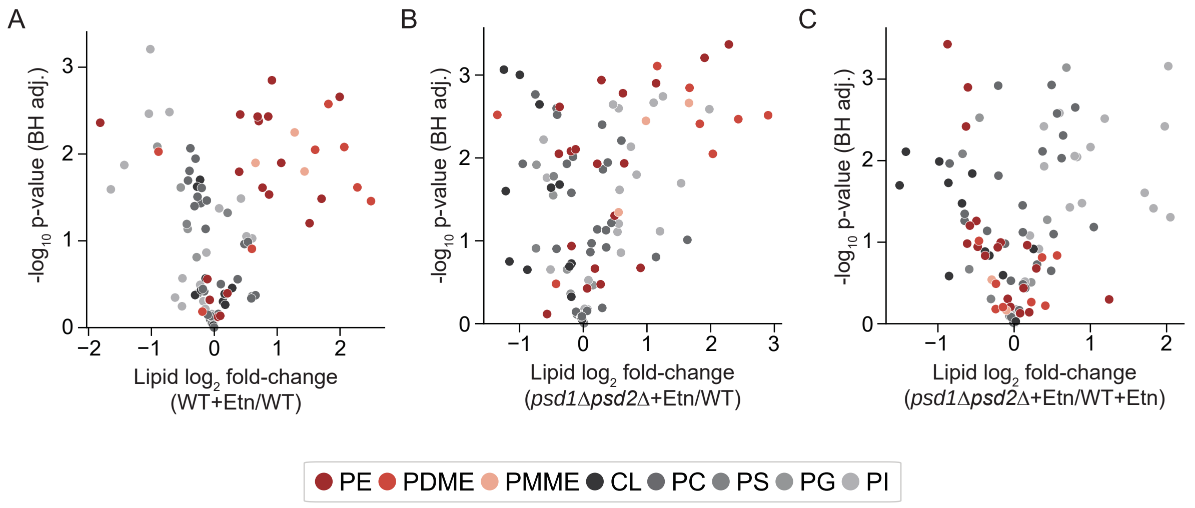

Supplementary Figure 1: Ethanolamine supplementation alters the cellular phospholipid profile.

(A) Relative whole cell abundances ($\log_2$ fold-change) of select phospholipid species for WT yeast supplemented with Etn compared to WT versus statistical significance. (B) Relative abundances ($\log_2$ fold-change) of select lipid species for *psd1Δpsd2Δ* yeast supplemented with Etn compared to WT versus statistical significance. (C) Relative abundances ($\log_2$ fold-change) of select lipid species for *psd1Δpsd2Δ* yeast supplemented with Etn compared to WT supplemented with Etn versus statistical significance. For all panels statistical significance is given as Benjamini-Hochberg adjusted p-value.

Supplementary Figure 2

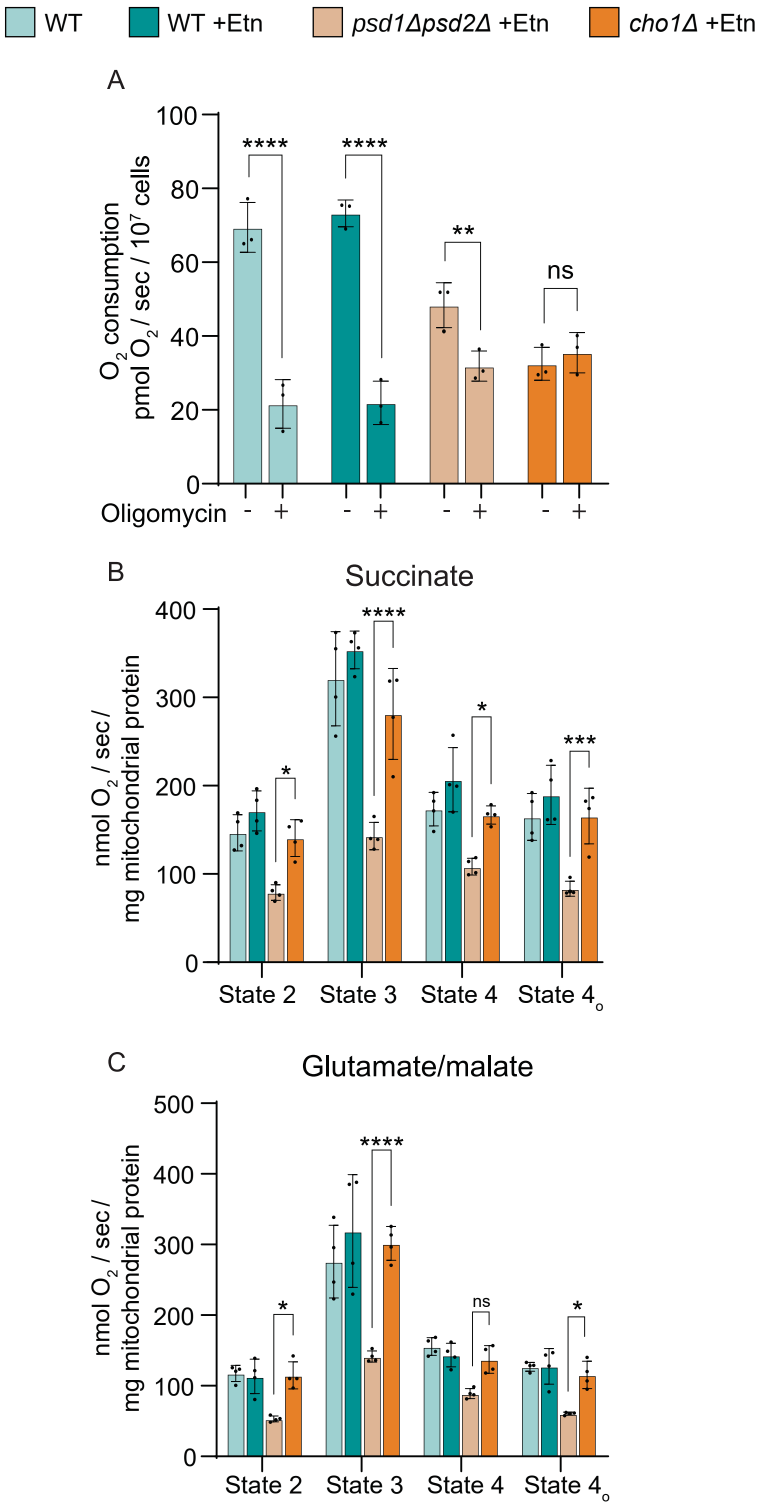

Supplementary Figure 2: Mitochondrial bioenergetics driven by alternative substrates in PS-lacking yeast.

(A) Cellular oxygen consumption rate of indicated yeast cells measured at 30°C. Cells were grown to early logarithmic phase and then treated with either oligomycin (3 μg/mL) or vehicle for 3 hours before performing respiration measurements. (B) States of mitochondrial respiration in the indicated yeast cells: Succinate-driven oxygen consumption (state 2), ADP-stimulated oxygen consumption (state 3), resting respiration after ADP consumption (state 4), and oligomycin induced resting respiration (state 4_o). Data are represented as mean ± SD (n = 4 biological replicates). (C) States of mitochondrial respiration in the indicated yeast cells: Glutamate/malate-driven oxygen consumption (state 2), ADP-stimulated oxygen consumption (state 3), resting respiration after ADP consumption (state 4), and oligomycin induced resting respiration (state 4_o). Data are represented as mean ± SD (n = 4 biological replicates).

****P < 0.0001, ***P < 0.001, **P < 0.01, *P < 0.05.

Supplementary Figure 3

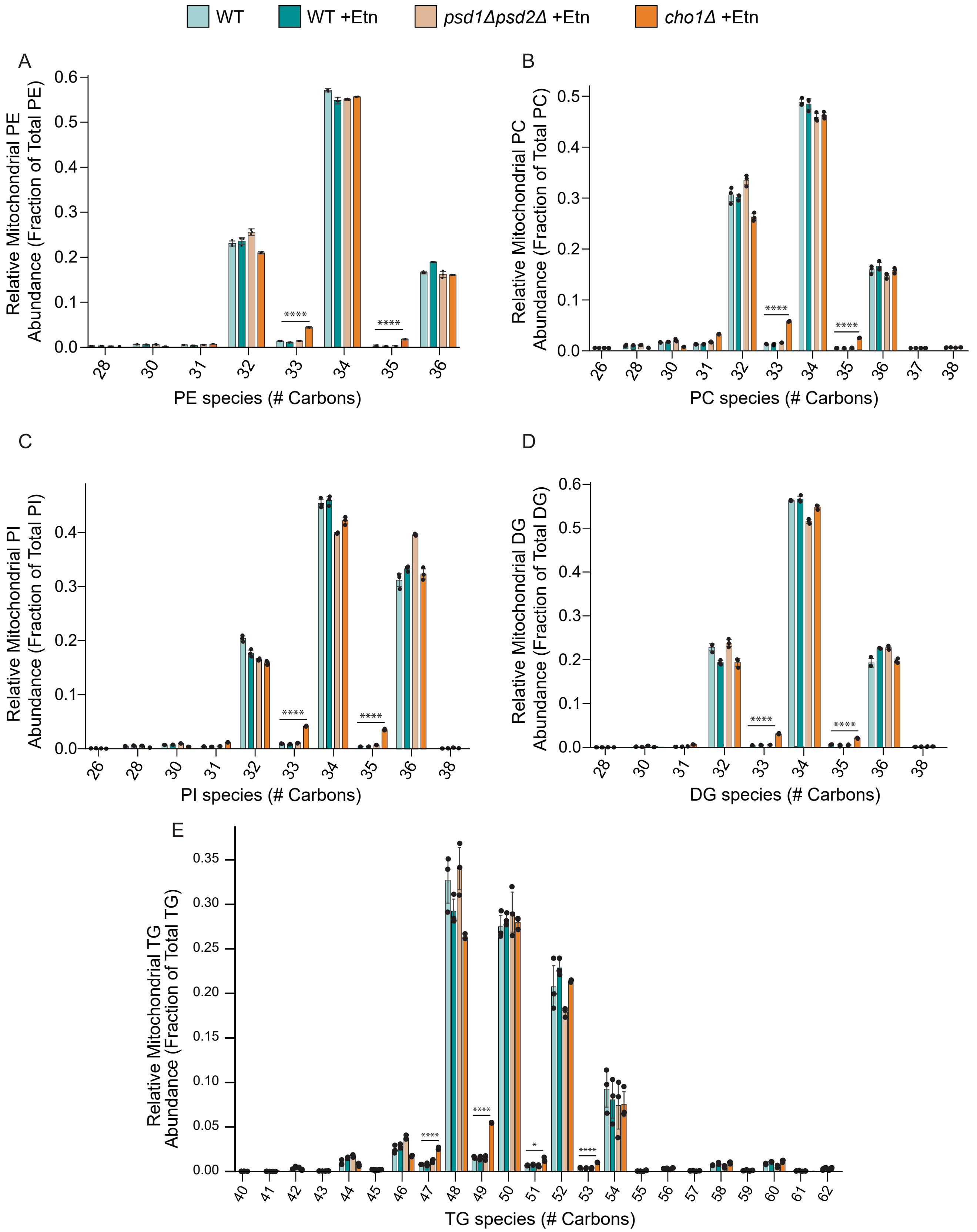

Supplementary Figure 3: Loss of PS leads to increased odd chain fatty acids in *cho1*Δ cells.

(A-E) Relative abundances of fatty acids with different chain lengths in mitochondrial (A) PE, (B) PC, (C) PI, (D) DG, and (E) TG species in WT, WT supplemented with Etn, *psd1*Δ*psd2*Δ supplemented with Etn, and *cho1*Δ supplemented with Etn yeast.

****P < 0.0001, *P < 0.05.

Supplementary Figure 4

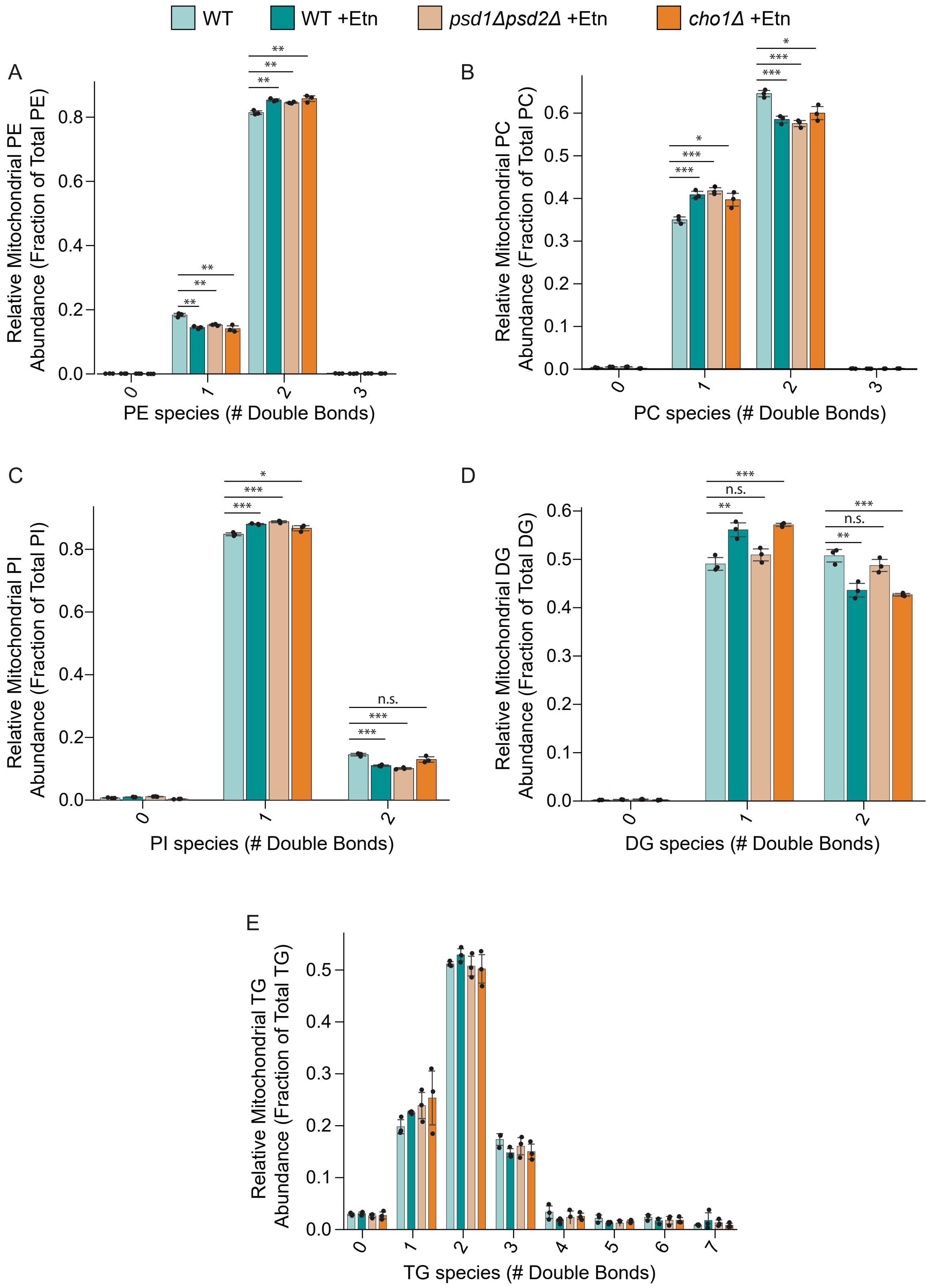

Supplementary Figure 4: Ethanolamine supplementation alters the mitochondrial phospholipid profile.

(A-E) Relative abundances of fatty acids with varying degrees of unsaturation in mitochondrial (A) PE, (B) PC, (C) PI, (D) DG, and (E) TG species in WT, WT supplemented with Etn, *psd1*Δ*psd2*Δ supplemented with Etn, and *cho1*Δ supplemented with Etn yeast.

***P < 0.001, **P < 0.01, *P < 0.05.

Supplementary Table 1: Yeast strains used in this study

Genotype	Source
BY4741 WT – *MATa, his3Δ1, leu2Δ0, met15Δ0, ura3Δ0*	Dr. M. L. Greenberg
BY4741 *psd1∆psd2∆* - *MATa, his3Δ1, leu2Δ0, met15Δ0, ura3Δ0, psd1∆::hphNT1 psd2∆:: kanMX4*	Iadarola et., al, 2021
BY4741 *cho1∆* - *MATa, his3Δ1, leu2Δ0, met15Δ0, ura3Δ0, cho1∆::kanMX4*	Open Biosystems

Supplementary Table 2: Sources of reagents used in this study

REAGENT or RESOURCE	SOURCE	IDENTIFIER
Antibodies		
Rabbit monoclonal Sdh2	Dr. Dennis Winge	None
Mouse monoclonal Rip1	Dr. Vincenzo Zara	None
Mouse monoclonal anti-Cox2	Abcam	Cat# ab110271
Mouse monoclonal anti-Cox4	Abcam	Cat# ab110272
Rabbit monoclonal anti-Atp2	Dr. Sharon Ackerman	None
Mouse monoclonal anti-Por1	Abcam	Cat# ab110326
Rabbit monoclonal anti-Tom70	Dr. Jan Brix	None
Rabbit monoclonal anti-Tim50	Dr. Jan Brix	None
Rabbit monoclonal anti-Tim44	Dr. Jan Brix	None
Chemicals		
Tetramethylrhodamine, Methyl Ester, Perchlorate (TMRM)	Invitrogen	Cat# T668
β-Nicotinamide adenine dinucleotide, reduced disodium salt hydrate	Sigma-Aldrich	Cat# N8129
Oligomycin from Streptomyces diastatochromogenes	Sigma-Aldrich	Cat# O4876
CCCP	Sigma-Aldrich	Cat# C2759
Adenosine 5′-diphosphate sodium salt	Sigma-Aldrich	Cat# A2754
Antimycin A from Streptomyces sp.	Sigma-Aldrich	Cat# A8674
Chloroform	Sigma-Aldrich	Cat# 650498
Methanol	Sigma-Aldrich	Cat# 34860
Ammonium hydroxide solution	Sigma-Aldrich	Cat# 221228

Supplementary Table 3: Compounds and Ions used in targeted LC-MS

Compound	Formula	Adduct	Precursor (m/z)	Quan Product Ion (m/z)	RT Time (min)
PS 32:1	C38H72NO10P	-H	732.4821	391.2253	5.46
PS 32:2	C38H70NO10P	-H	730.4665	391.2253	6.08
PS 34:1	C40H76NO10P	-H	760.5134	389.2098	5.14
PS 34:2	C40H74NO10P	-H	758.4978	389.2098	5.49
PS 36:2	C42H78NO10P	-H	786.5291	417.2403	6.12
