# Supplementary material for: Mitochondrial dysfunction and lipid dysregulation in yeast lacking phosphatidylserine
Source: Mol Biol Cell. 2025 Aug 13;36(10):ar121. doi: 10.1091/mbc.E25-03-0128 (PMC12444905; doi:10.1091/mbc.E25-03-0128)
Supplement: Supplementary file 2 [file reviewer_reportE25-03-0128.pdf]

Mitochondrial dysfunction and lipid dysregulation in yeast lacking phosphatidylserine

Alaumy Joshi, Zakery Baker, Rachel Stanfield, Dimitris Kalafatis, David Pagliarini, and Vishal Gohil

Corresponding author(s): Vishal Gohil, Texas A&M University

Review Timeline:

Submission Date:	2025-03-23
Editorial Decision:	2025-04-22
Revision Received:	2025-07-28
Accepted:	2025-08-01

Editor-in-Chief: John Aitchison

Transaction Report:

(Note: With the exception of the correction of typographical or spelling errors that could be a source of ambiguity, letters and reports are not edited. The original formatting of letters and referee reports may not be reflected in this compilation.)

RE: Manuscript #E25-03-0128
TITLE: Mitochondrial dysfunction and lipid dysregulation in yeast lacking phosphatidylserine

Dear Dr. Gohil:
thank you very much for submitting your work to MBoC. Your work has been evaluated by two expert reviewers who both are positive about your study, but suggest to further characterize phospholipids, and particular their acyl chains, in your newly constructed yeast models, to determine respiratory rates with more substrates, and to clarify additional minor points. These new analyses will further strengthen your elegant study. We are happy to invite you to submit a revised version of your study, along with a detailed description of how you addressed the reviewer's comments.
Thanks again for submitting your work to MBoC and we are looking forward to receiving your revised manuscript.

Sincerely,

Martin Ott
Monitoring Editor
Molecular Biology of the Cell

Dear Dr. Gohil,

The review of your manuscript, referenced above, is now complete. The Monitoring Editor has decided that your manuscript is not acceptable for publication at this time, but may be deemed acceptable after specific revisions are made, as described in the Monitoring Editor's decision letter above and the reviewer comments below.

A reminder: Please do not contact the Monitoring Editor directly regarding your manuscript. If you have any questions regarding the review process or the decision, please contact the MBoC Editorial Office (mboc@ascb.org).

When submitting your revision include a rebuttal letter that details, point-by-point, how the Monitoring Editor's and reviewers' comments have been addressed. (The file type for this letter must be "rebuttal letter"; do not include your response to the Monitoring Editor and reviewers in a "cover letter.") Please bear in mind that your rebuttal letter will be published with your paper if it is accepted, unless you have opted out of publishing the review history.

Authors are allowed 90 days to submit a revision. If this time period is inadequate, please contact us at mboc@ascb.org.

Revised manuscripts are assigned to the original Monitoring Editor whenever possible. However, special circumstances may preclude this. Also, revised manuscripts are often sent out for re-review, usually to the original reviewers when possible. The Monitoring Editor may solicit additional reviews if it is deemed necessary to render a completely informed decision.

In preparing your revised manuscript, please follow the instruction in the Information for Authors (www.molbiolcell.org/info-for-authors). In particular, to prepare for the possible acceptance of your revised manuscript, submit final, publication-quality figures with your revision as described.

To submit the rebuttal letter, revised manuscript, and figures, use this link: Link Not Available

MBoC offers the option to publish your work with immediate open access. Open access can increase the discoverability and usability of your research. If you would like to publish your paper with immediate open access but did not select this option during initial submission, please contact the editorial office at mbc@ascb.org.

Authors of Articles and Brief Communications are also encouraged to create a short video abstract to accompany their article when it is published. These video abstracts, known as Science Sketches, are up to 2 minutes long and will be published on YouTube and then embedded in the article abstract. Science Sketch Editors on the MBoC Editorial Board will guide you as you prepare your video. Information about how to prepare and submit a video abstract is available at www.molbiolcell.org/science-sketches. Please contact mboc@ascb.org if you are interested in creating a Science Sketch.

Sign up for MBoC's new content alerts here https://tinyurl.com/3bcsyk3e

Thank you for submitting your manuscript to MBoC. We look forward to receiving your revised paper.

Sincerely,

Eric Baker
Journal Production Manager
MBoC Editorial Office
mbc@ascb.org

Reviewer #1 (Remarks to the Author):

In the manuscript by Joshi et al, the authors developed a clever strategy to dissect the role that the low abundance phospholipid phosphatidylserine (PS) plays in mitochondrial function. The overall approach based on the comparison of ethanolamine supplemented cho1 and psd1psd2 mutants is well thought out and allows to relate the observed phenotypes to the specific lack of PS. The authors show that in absence of PS, cells grow poorly in respiratory media and have a slightly reduced respiratory capacity. However, OXPHOS complex levels in isolated mitochondria are unaffected and mitochondrial respiration is significantly increased. Is mitochondrial content/mass in these cells comparable to controls? This may be relevant also because mutations in pdr1 and 2 have been linked to alterations in vacuolar morphology and mitophagy. Still in relation to the apparent discrepancy between cellular and mitochondrial respiration, to confirm the high degree of proton leak in cho1 mutant cells, leak/state 4 respiration could be measured in whole cells upon addition of oligomycin. Additionally, while whole cells utilize a mix of intracellular substrates, only NADH-driven respiration was measured in mitochondria. Is the behavior similar when using different respiratory substrates? Lastly, the authors show that loss of PS induces an increase in odd-chain fatty acids, which are known to increase membrane fluidity. Notably, an increase in mitochondrial membrane unsaturated lipids, which also increase membrane fluidity, has been shown to increase respiratory capacity (although no state 4/leak respiration was measured in the study. DOI: 10.1126/science.aat7925). Is the content of mitochondrial unsaturated lipids also changed in consequence of PS alterations? Is mitochondrial membrane fluidity affected?

Reviewer #2 (Remarks to the Author):

This interesting study by Joshi et. al describes novel roles for phosphatidylserine (PS) metabolism in shaping mitochondrial structure and function in budding yeast. PS is not an abundant lipid of mitochondria but is a crucial precursor to PE biosynthesis which occurs in the inner membrane via the PS decarboxylase Psd1. The roles of PS in mitochondria have traditionally been difficult to study as genetic deletion of PS synthase (cho1) results in pleiotropic effects on the synthesis of major mitochondrial phospholipids, such as PC and PE. The authors circumvent these effects through supplementation of cho1 knockouts with ethanolamine which restores PE levels despite the loss of PS. The authors show that the loss of PS leads to defective growth on respiratory media and reduced membrane potential without altering the assembly or abundance of electron transport chain proteins, which the authors attribute to defects in mitochondrial membrane integrity. Surprisingly, the authors observe increased levels of odd-chained fatty acids in both whole cell and mitochondrial lipids in PS-deficient cells, which they propose could contribute to defective membrane property.

Overall, while this study presents a useful genetic model for interrogating the roles of PS in mitochondrial physiology, additional experiments and clarifications are necessary to properly interpret the results from this study. I have summarized these points below:

Major:

1) While the lipidomics analysis conducted in this study are thorough, I have major issues with their representation. In Figure 2A-C, it is very difficult to interpret the color differences between PC/PS/PG and PI, making it difficult to assess relative changes in abundances of these species, which are crucial for proper interpretation of this figure and the overall genetic model.

For clarity, the authors should plot the total abundances of each of these phospholipid species as a fraction of the total mitochondrial phospholipids, rather than depicting individual fold changes to select phospholipid sub-species. Furthermore, an identical analysis of mitochondrial phospholipid abundances should be presented for cho1+Etn in this figure as this is critical for proper understanding of the study and cross comparisons between data presented in Figure 5 and Figure 2 are difficult.

Without knowing absolute changes in mitochondrial PE levels in cho1+Etn, it is not possible to exclude that decreased in total mitochondrial PE could be the primary cause of respiratory dysfunction in cho1+Etn rather than loss of PS.

2) While this study focuses on potential contributions of PS in mitochondrial function, it also presents an intriguing opportunity to compare the contributions of ER-synthesized and mitochondria-synthesized PE in shaping bioenergetics. The authors could accomplish this by further delving into the compositions of PE within the mitochondria of psd1psd2+Etn and cho1+Etn.

While decreases in specific unsaturated PE species are shown in Figure 2D for psd1psd2+Etn, in Figure 5B, it appears that select saturated PE species are decreasing in cho1+Etn, and that unsaturated PE species increase. Could this suggest that in

the absence of PS more unsaturated PE is selectively imported into mitochondria? Previous studies have shown that unsaturated PS is selectively imported at contact sites for mitochondrial PE synthesis. The authors should comment on this.

To better understand changes in acyl chain composition, the authors should consider plotting the %acyl chain unsaturation or double bond index rather than the abundances of selected saturated and unsaturated phospholipid species.

3) The observation of changes in odd-chained fatty acid chains on phospholipids is intriguing. However, what are the total levels of these odd-chained mitochondrial phospholipids compared to their even counterparts especially in cho1+Etn? What is the reference population that the z-score is comparing to in Figure 5A and B? While there seem to be increases in odd-chained phospholipid species without knowing the reference population it is not possible to infer their roles on bulk membrane property (if they are in very low abundance).

Minor points:

1) Does Figure 5C+D depict fold changes of cho1KO vs. WT or cho1+Etn vs. WT.
2) What is the total abundance of PDME and PMME species compared to total PE within the mitochondria? Why do the authors think that these PE->PC methylation intermediates are present within mitochondria?
3) Since this study contains analysis of numerous ER-derived lipids within mitochondria- it would be useful to quantify the extent of ER contamination within isolated mitochondria from WT and mutant strains.

Reviewer #1 (Remarks to the Author):

In the manuscript by Joshi et al, the authors developed a clever strategy to dissect the role that the low abundance phospholipid phosphatidylserine (PS) plays in mitochondrial function. The overall approach based on the comparison of ethanolamine supplemented cho1 and psd1psd2 mutants is well thought out and allows to relate the observed phenotypes to the specific lack of PS.

The authors show that in absence of PS, cells grow poorly in respiratory media and have a slightly reduced respiratory capacity. However, OXPHOS complex levels in isolated mitochondria are unaffected and mitochondrial respiration is significantly increased. Is mitochondrial content/mass in these cells comparable to controls? This may be relevant also because mutations in pdr1 and 2 have been linked to alterations in vacuolar morphology and mitophagy.

We have now measured mitochondrial mtDNA copy number, a reliable indicator of mitochondrial mass, and found no significant difference in the mtDNA copy number in any of mutants, including the PS-deficient *cho1Δ* (Figure below and Figure 3C in the revised draft). We used growth in glucose (fermentable media, which is known to suppress mitochondrial biogenesis) and ethidium bromide treatment (which is known to reduce mtDNA copy number) as controls in this experiment (see Figure below).

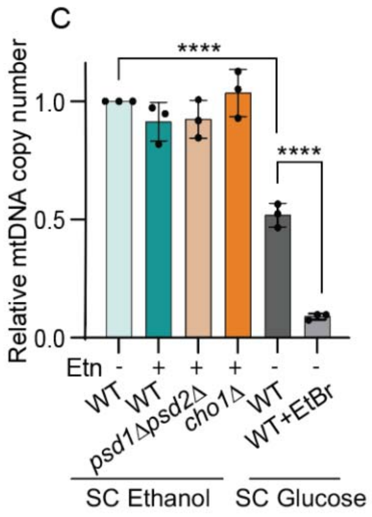

Still in relation to the apparent discrepancy between cellular and mitochondrial respiration, to confirm the high degree of proton leak in cho1 mutant cells, leak/state 4 respiration could be measured in whole cells upon addition of oligomycin.

We thank the reviewer for this excellent suggestion. We have now measured the whole cell respiration upon the addition of oligomycin and found that unlike wild-type or the *psd1Δpsd2Δ* mutant, oligomycin treatment does not decrease respiration in *cho1Δ* cells (see Figure below, and Supplementary Figure 2A in the revised manuscript). This almost complete lack of ATP-coupled respiration is consistent with a high degree of proton leak.

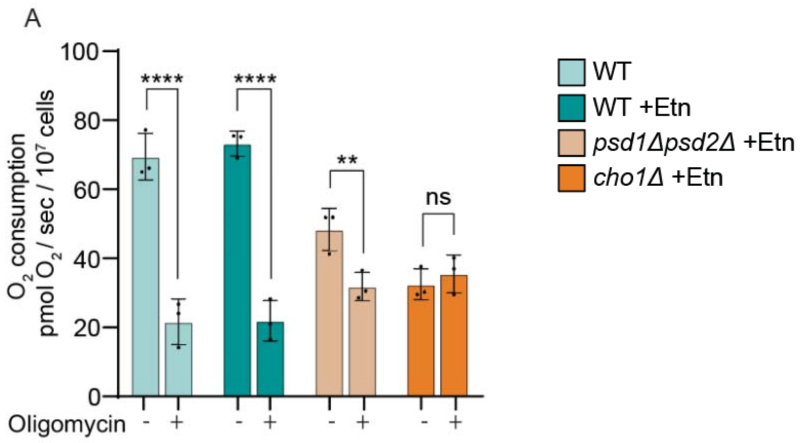

Additionally, while whole cells utilize a mix of intracellular substrates, only NADH-driven respiration was measured in mitochondria. Is the behavior similar when using different respiratory substrates?

We have now measured mitochondrial respiration using different substrates, including glutamate/malate and succinate and found similar trend in respiration in these substrates (See Figures below, and Supplementary Figure 2B-C). As compared to *psd1Δpsd2Δ* mutant, we found increased respiration in *cho1Δ* mitochondria in all four states (State 2, 3, 4, and 4o), consistent with what we had observed with NADH as a respiratory substrate. Though, we note that *cho1Δ* mitochondria exhibited more coupled respiration with these substrates as compared to NADH, with their respiration rates more comparable to wild-type conditions. We have now described these results in the revised text.

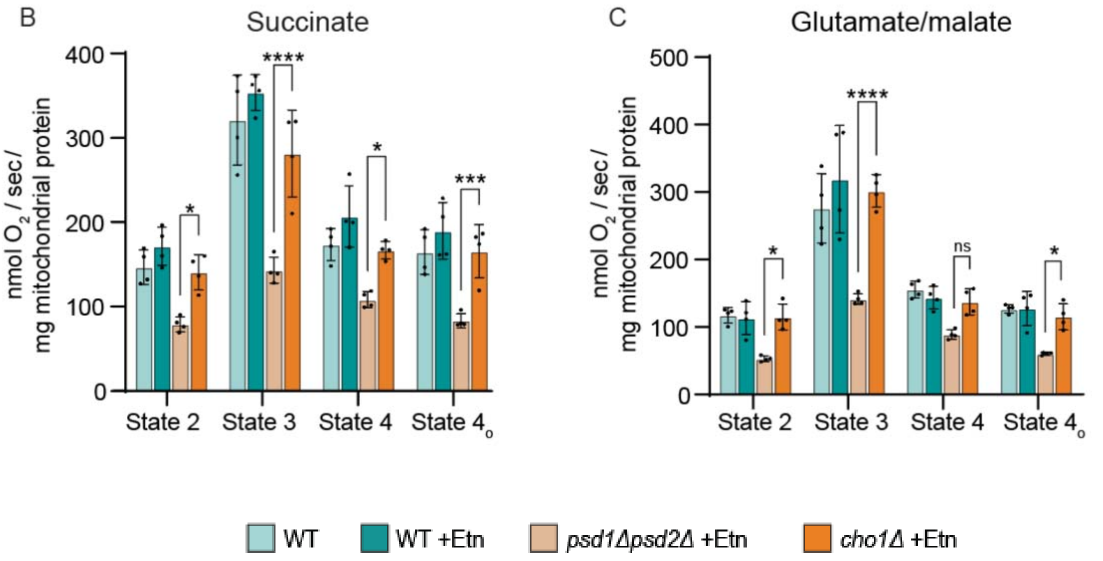

Lastly, the authors show that loss of PS induces an increase in odd-chain fatty acids, which are known to increase membrane fluidity. Notably, an increase in mitochondrial membrane unsaturated lipids, which also increase membrane fluidity, has been shown to increase respiratory capacity (although no state 4/leak respiration was measured in the study. DOI: 10.1126/science.aat7925). Is the content of mitochondrial unsaturated lipids

`also changed in consequence of PS alterations? Is mitochondrial membrane fluidity affected?`

We again thank the reviewer for bringing this important point to our attention. We now discuss papers that highlight the influence of fatty acyl composition on the biophysical properties of mitochondrial membranes (Venkatraman et al., 2024, Budin et al., Science 2018). Unlike a specific increase in odd chain fatty acyl species in *cho1Δ* mutant, we do not see a clear trend of increased unsaturation in these cells. The alteration in the saturation status of fatty acyl species is more a function of Etn supplementation rather than PS deficiency (See Figure below, and Supplementary Figure 4).

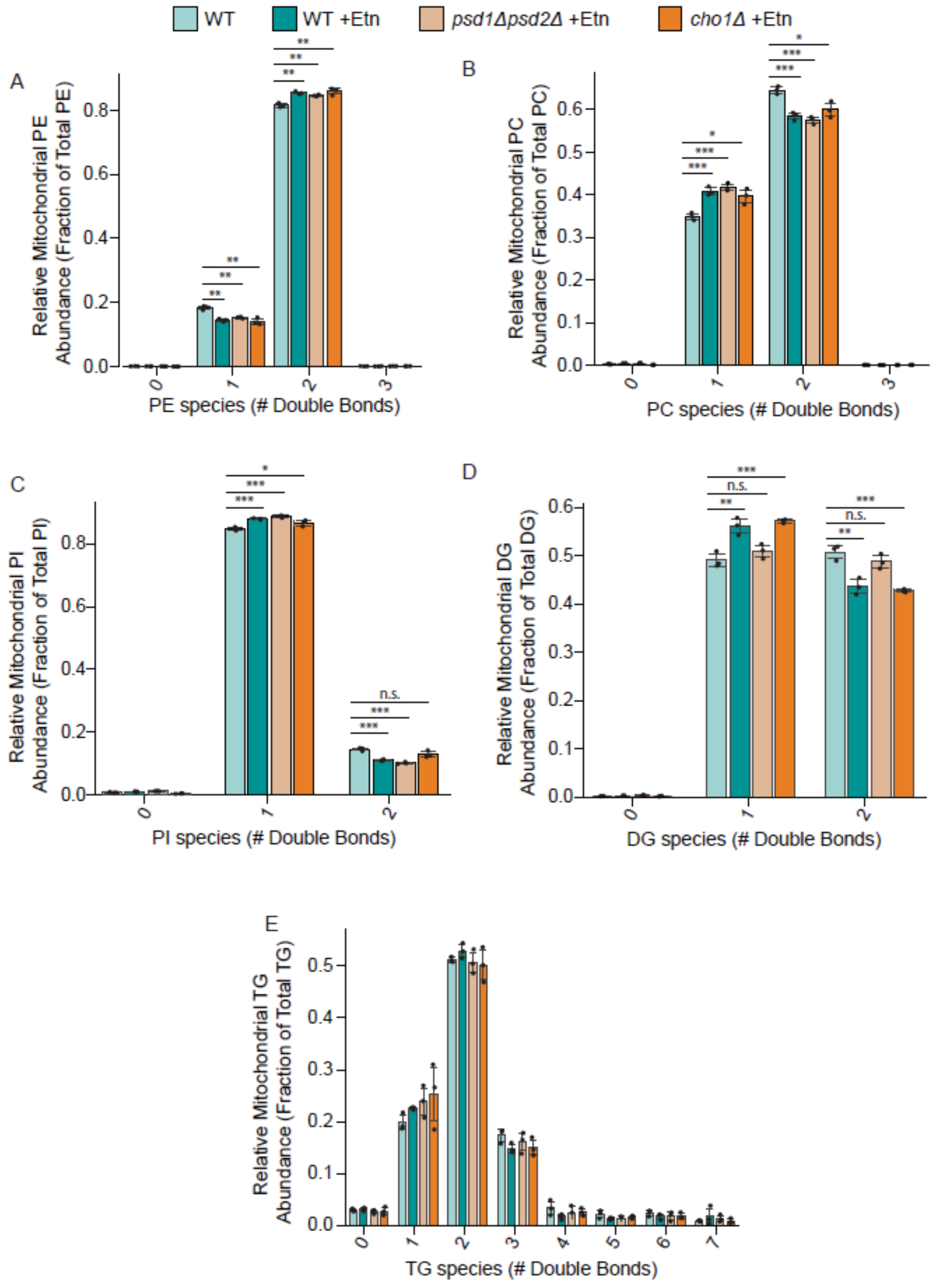

Supplemental Figure 4

Reviewer #2 (Remarks to the Author):

This interesting study by Joshi et. al describes novel roles for phosphatidylserine (PS) metabolism in shaping mitochondrial structure and function in budding yeast. PS is not an abundant lipid of mitochondria but is a crucial precursor to PE biosynthesis which occurs in the inner membrane via the PS decarboxylase Psd1. The roles of PS in mitochondria have traditionally been difficult to study as genetic deletion of PS synthase (cho1) results in pleiotropic effects on the synthesis of major mitochondrial phospholipids, such as PC and PE. The authors circumvent these effects through supplementation of cho1 knockouts with ethanolamine which restores PE levels despite the loss of PS. The authors show that the loss of PS leads to defective growth on respiratory media and reduced membrane potential without altering the assembly or abundance of electron transport chain proteins, which the authors attribute to defects in mitochondrial membrane integrity. Surprisingly, the authors observe increased levels of odd-chained fatty acids in both whole cell and mitochondrial lipids in PS-deficient cells, which they propose could contribute to defective membrane property.

Overall, while this study presents a useful genetic model for interrogating the roles of PS in mitochondrial physiology, additional experiments and clarifications are necessary to properly interpret the results from this study. I have summarized these points below:

Major:

1) While the lipidomics analysis conducted in this study are thorough, I have major issues with their representation. In Figure 2A-C, it is very difficult to interpret the color differences between PC/PS/PG and PI, making it difficult to assess relative changes in abundances of these species, which are crucial for proper interpretation of this figure and the overall genetic model.

We have now changed the color scheme so that it is easier to tell all the different phospholipids apart (See Figures below, and Figures 2A-D in the revised manuscript). We have also added *cho1Δ*+Etn/WT+Etn volcano plot as panel D, which lacks PS.

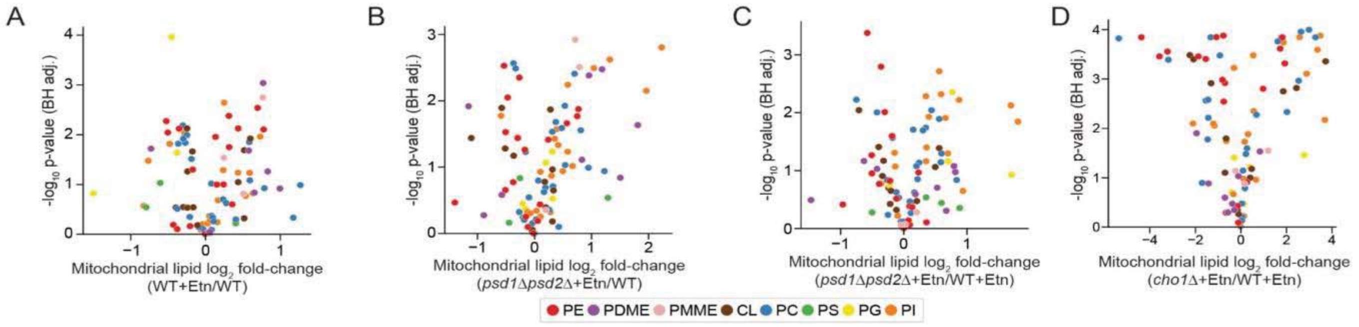

For clarity, the authors should plot the total abundances of each of these phospholipid species as a fraction of the total mitochondrial phospholipids, rather than depicting individual fold changes to select phospholipid sub-species. Furthermore, an identical analysis of mitochondrial phospholipid abundances should be presented for cho1+Etn in this figure as this is critical for proper understanding of the study and cross comparisons between data presented in Figure 5 and Figure 2 are difficult.

Since we only have relative abundance values (not absolute abundance concentrations) for each lipid species, it precludes us from comparing across different lipid classes as each class can ionize differently which will affect its peak intensity. Though, we can still compare

a phospholipid class across different samples. To accomplish this, we have combined the intensities for each of the PE, CL, and PC species in every condition (see Figures below, and Figures 2H-J in the revised manuscript). As requested by this reviewer, we have also added data from the *cho1Δ*+Etn sample into these figure panels.

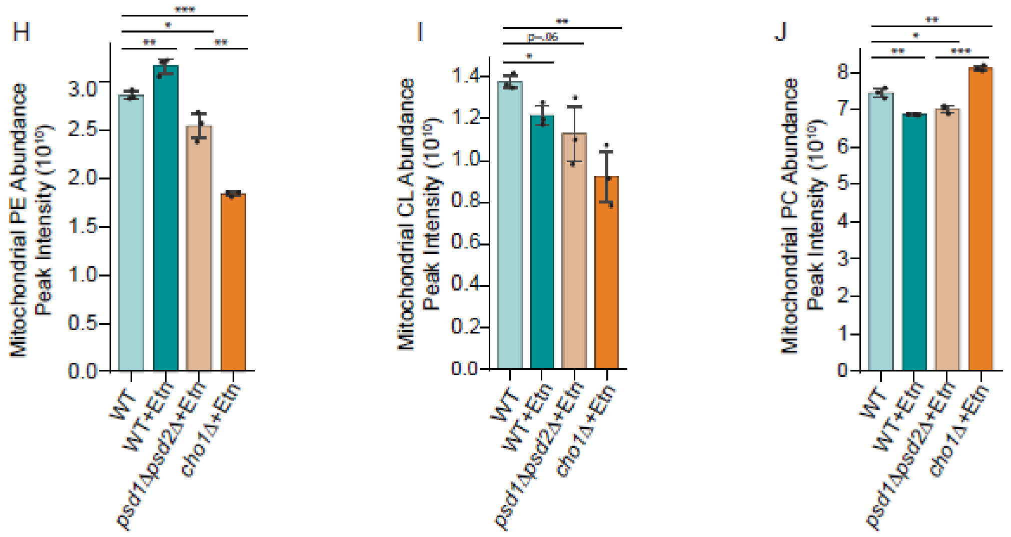


```
Without knowing absolute changes in mitochondrial PE levels in cho1+Etn, it is
not possible to exclude that decreased in total mitochondrial PE could be the
primary cause of respiratory dysfunction in cho1+Etn rather than loss of PS.
```

The reviewer brings up an excellent point. Indeed, upon calculating the total mitochondrial PE abundance (See Figure above, and Figure 2H in the revised manuscript), we observe lower levels of mitochondrial PE in the *cho1Δ*+Etn compared to the *psd1Δpsd2Δ*+Etn. This observation suggests that bioenergetic defects in *cho1Δ*+Etn cells could stem from this decrease in PE. We have now modified our text to account for this possibility. Specifically, we discuss the potential requirement of PS in the import of Kennedy pathway-derived PE into mitochondria. This new analysis offers another potential mechanism by which PS impacts mitochondrial respiratory function.

```
2) While this study focuses on potential contributions of PS in mitochondrial
   function, it also presents an intriguing opportunity to compare the
   contributions of ER-synthesized and mitochondria-synthesized PE in shaping
   bioenergetics. The authors could accomplish this by further delving into
   the compositions of PE within the mitochondria of psd1psd2+Etn and
   cho1+Etn.
```

The reviewer is correct, comparing mitochondrial PE species in cells lacking mitochondrial PE synthesis (*psd1Δpsd2Δ*+Etn and *cho1Δ*+Etn) with that of WT and WT+Etn, can tell us the contribution of the ER-synthesized and mitochondria-synthesized PE in mitochondrial bioenergetics. To this end, we analyzed the composition of mitochondrial PE species in all four conditions by combining the intensities of PE species based on the number of double bonds. We noticed that Etn supplementation, which stimulates the Kennedy pathway in the ER, leads to a small but significant increase in PE species with two double bonds with a concomitant decrease in species with a single double bond in all yeast strains tested (WT+Etn, *psd1Δpsd2Δ*+Etn and *cho1Δ*+Etn) (See Figure below and Supplementary Figure 4A). This new figure when combined with our original Figure 2D (which is now Figure 2E), where we provide levels of individual PE species, together identifies Kennedy-

pathway specific changes. Though, we note that there are relatively minor alterations in overall mitochondrial PE composition upon stimulation of the Kennedy pathway. As noted in response to this reviewer's next comments, the most striking *cho1Δ*-specific changes we observe are in the odd chain fatty acid levels.

While decreases in specific unsaturated PE species are shown in Figure 2D for psd1psd2+Etn, in Figure 5B, it appears that select saturated PE species are decreasing in cho1+Etn, and that unsaturated PE species increase. Could this suggest that in the absence of PS more unsaturated PE is selectively imported into mitochondria? Previous studies have shown that unsaturated PS is

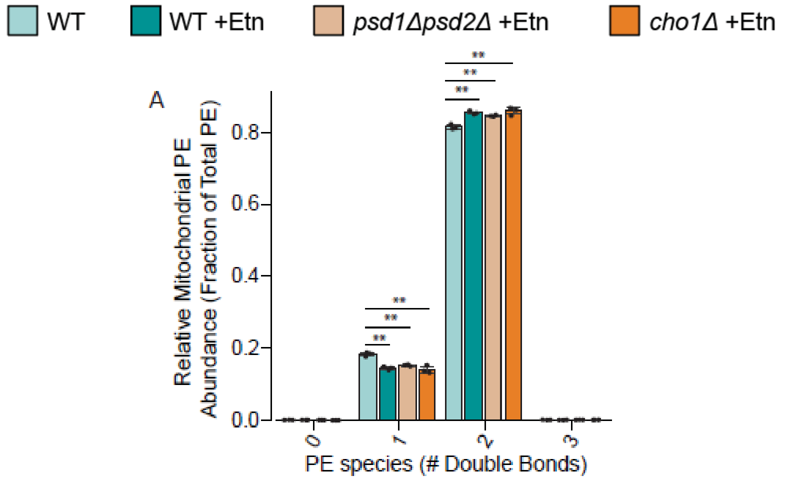

selectively imported at contact sites for mitochondrial PE synthesis. The authors should comment on this.

The increase in unsaturated PE species we observe in *cho1Δ*+Etn is mainly in the odd chain fatty acids (Revised Figure 5A and below), which is consistent with an overall increase in odd chain fatty acid levels in *cho1Δ*+Etn. However, since we observe a consistent increase in unsaturated PE species (shown above and new Supplementary Figure 4A) in all strains treated with Etn, we think that the stimulation of the Kennedy pathway is the primary driver of increased unsaturated PE species in mitochondria.

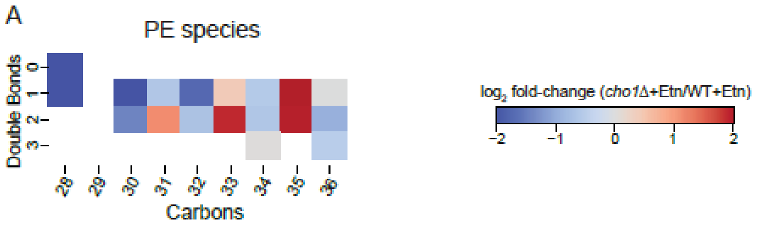

To better understand changes in acyl chain composition, the authors should consider plotting the %acyl chain unsaturation or double bond index rather than the abundances of selected saturated and unsaturated phospholipid species.

We have now plotted the data accordingly (Supplementary Figure 4 and below). We have also plotted heatmaps showing relative acyl chain unsaturation in terms of the number of double bonds in different lipid (PE, PC, PI, DG, TG) species compared to the WT + Etn (Figure 5 and below).

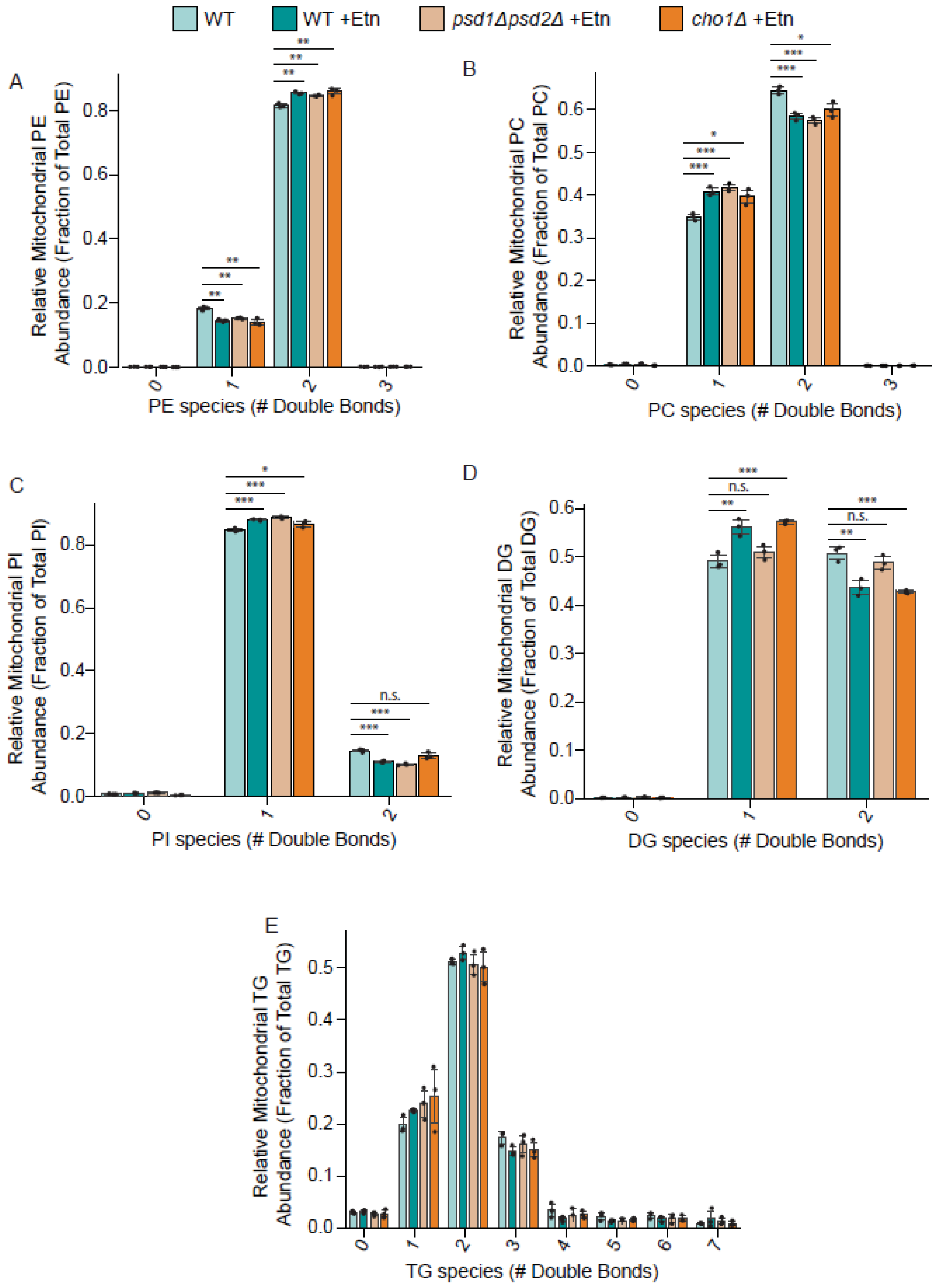

Supplementary Figure 4

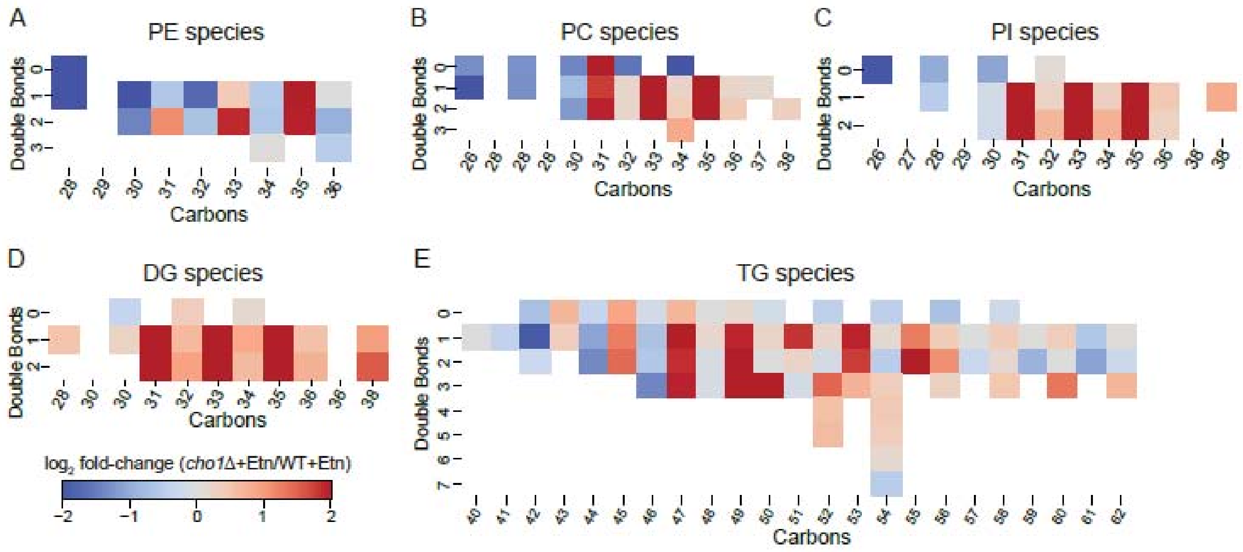

Figure 5

3) The observation of changes in odd-chained fatty acid chains on phospholipids is intriguing. However, what are the total levels of these odd-chained mitochondrial phospholipids compared to their even counterparts especially in cho1+Etn? What is the reference population that the z-score is comparing to in Figure 5A and B? While there seem to be increases in odd-chained phospholipid species without knowing the reference population it is not possible to infer their roles on bulk membrane property (if they are in very low abundance).

This is an important point raised by the reviewer. We have now updated our Figure 5 (see above) and have presented data where total abundance of odd chain fatty-containing lipid class are shown in relation to their even counter parts (Supplementary Figure 3 and below). As shown here the abundance of odd chain tails in *cho1Δ*+Etn is relatively small when compared to the abundance of even chain tails, but it is robust and seen across all lipid classes.

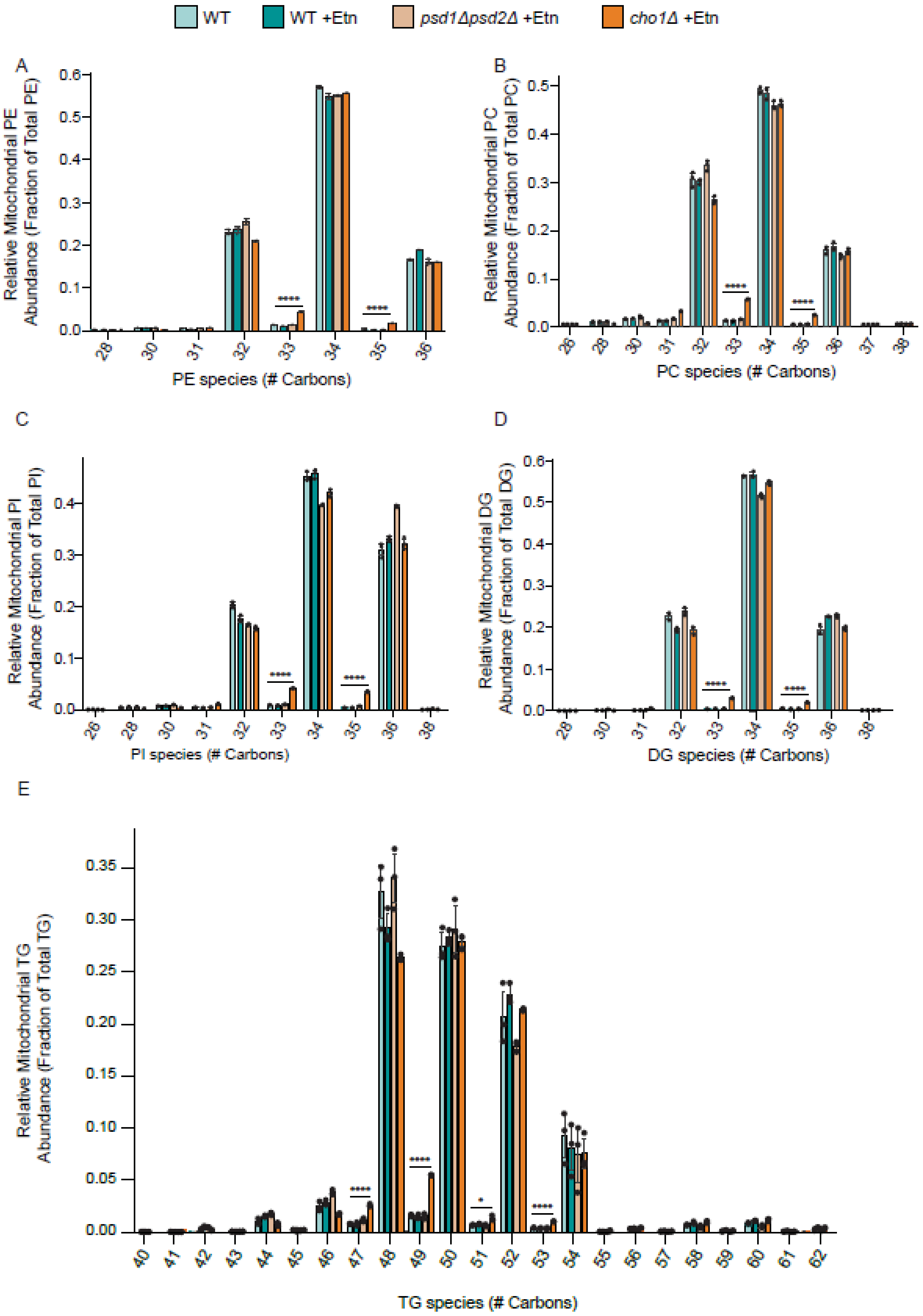

Supplementary Figure 3

Minor points:

1) Does Figure 5C+D depict fold changes of cho1KO vs. WT or cho1+Etn vs. WT.

This panel has been updated to the heatmaps (Figures 5A-E in the revised manuscript) which use the correct *cho1Δ*+Etn/WT+Etn comparison.

2) What is the total abundance of PDME and PMME species compared to total PE within the mitochondria? Why do the authors think that these PE->PC methylation intermediates are present within mitochondria?

While the intensities of the N-methyl-PE species that we detected was much lower than PE species (50-100x lower in combined detected intensities), we only have relative quantification of the individual lipid species and therefore we cannot make direct comparisons between the lipid classes as mentioned above. Any N-methyl-PE species would be from the ER contamination from Mito:ER contact sites and mitochondrial associated membranes (MAMs) as shown below in response to the next comment.

3) Since this study contains analysis of numerous ER-derived lipids within mitochondria- it would be useful to quantify the extent of ER contamination within isolated mitochondria from WT and mutant strains.

To assess the extent of ER contamination within isolated mitochondria, we have now measured markers of mitochondria, ER, and cytosol in crude versus density gradient purified mitochondria by western blotting (see Figure below). The presence of ER-marker protein in our density gradient purified mitochondrial isolates ('Pure') suggest ER-contamination, which is most likely due to MAMs. We have now discussed this in the manuscript text.

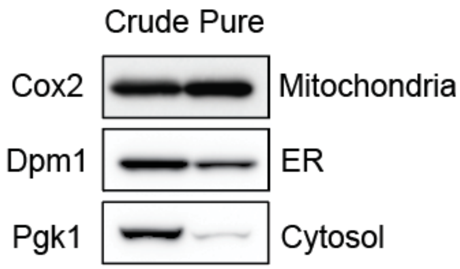

RE: Manuscript #E25-03-0128R
TITLE: "Mitochondrial dysfunction and lipid dysregulation in yeast lacking phosphatidylserine"

Dear Dr. Gohil:

thanks a lot for sending the revised version of your manuscript. After carefully reading the rebuttal letter and your manuscript, I am pleased to accept your manuscript for publication in Molecular Biology of the Cell.

Sincerely,
Martin Ott
Monitoring Editor
Molecular Biology of the Cell

Dear Dr. Gohil:

Congratulations on the acceptance of your manuscript! Thank you for publishing your work in Molecular Biology of the Cell (MBoC).

Within 10 days, an unedited PDF of your manuscript will be published on MBoC in Press, an early-release journal version. The date your manuscript appears on this site is the official publication date.

Your copyedited and typeset manuscript will be scheduled for publication in the next available issue of MBoC. Once your paper is ready for review, our production team will notify you. In the summer of 2024, our production provider will introduce an online proofing solution, which will streamline the process of checking and validating author changes. This will result in improved typesetting quality, enhanced digital content, and a faster overall process.

We invite you to submit images related to your accepted manuscript to be considered for the journal cover.
Cover images must contain color, TIF Format
Size image at 7.4" wide x 5.85" deep
RGB and CMYK color (please submit two separate files, one in each format)
300 dpi resolution

Please also send a 1-paragraph description of the image that a general audience could understand. Include with the caption the name and institution of the individual(s) the image should be attributed to. Please provide this description in a Word file. You can send the file via email or by your favorite file transfer service to mboc@ascb.org.

Authors of Articles and Brief Communications are also encouraged to create a short video abstract to accompany their article when it is published. These video abstracts, known as Science Sketches, are up to 2 minutes long and will be published on YouTube and then embedded in the article abstract. Science Sketch Editors on the MBoC Editorial Board will guide you as you prepare your video. Information about how to prepare and submit a video abstract is available at www.molbiolcell.org/science-sketches. Please contact mboc@ascb.org if you are interested in creating a Science Sketch.

We look forward to publishing your paper in MBoC.
Sign up for MBoC's new content alerts here https://tinyurl.com/3bcsyk3e

Sincerely,

Eric Baker
Journal Production Manager
MBoC Editorial Office
mbc@ascb.org
